# Dissociation of tau pathology and neuronal hypometabolism within the ATN framework of Alzheimer’s disease

**DOI:** 10.1038/s41467-022-28941-1

**Published:** 2022-03-21

**Authors:** Michael Tran Duong, Sandhitsu R. Das, Xueying Lyu, Long Xie, Hayley Richardson, Sharon X. Xie, Paul A. Yushkevich, Alzheimer’s Disease Neuroimaging Initiative (ADNI), Michael Weiner, Paul Aisen, Ronald Petersen, Clifford R. Jack, William Jagust, John Q. Trojanowki, Arthur W. Toga, Laurel Beckett, Robert C. Green, Andrew J. Saykin, John C. Morris, Leslie M. Shaw, Enchi Liu, Tom Montine, Ronald G. Thomas, Michael Donohue, Sarah Walter, Devon Gessert, Tamie Sather, Gustavo Jimenez-Maggiora, Danielle Harvey, Matthew Bernstein, Nick Fox, Paul Thompson, Norbert Schuff, Charles DeCarli, Bret Borowski, Jeff Gunter, Matt Senjem, Prashanthi Vemuri, David Jones, Kejal Kantarci, Chad Ward, Robert A. Koeppe, Norm Foster, Eric M. Reiman, Kewei Chen, Chet Mathis, Susan Landau, Nigel J. Cairns, Erin Householder, Lisa Taylor-Reinwald, Virginia M-Y Lee, Magdalena Korecka, Michal Figurski, Karen Crawford, Scott Neu, Tatiana M. Foroud, Li Shen, Kelley Faber, Sungeun Kim, Kwangsik Nho, Zaven Khachaturian, Richard Frank, Peter J. Snyder, Susan Molchan, Jeffrey Kaye, Joseph Quinn, Betty Lind, Raina Carter, Sara Dolen, Lon S. Schneider, Sonia Pawluczyk, Mauricio Beccera, Liberty Teodoro, Bryan M. Spann, James Brewer, Helen Vanderswag, Adam Fleisher, Judith L. Heidebrink, Joanne L. Lord, Sara S. Mason, Colleen S. Albers, David Knopman, Kris Johnson, Rachelle S. Doody, Javier Villanueva-Meyer, Munir Chowdhury, Susan Rountree, Mimi Dang, Yaakov Stern, Lawrence S. Honig, Karen L. Bell, Beau Ances, Maria Carroll, Sue Leon, Mark A. Mintun, Stacy Schneider, Angela Oliver, Randall Griffith, David Clark, David Geldmacher, John Brockington, Erik Roberson, Hillel Grossman, Effie Mitsis, Leyla deToledo-Morrell, Raj C. Shah, Ranjan Duara, Daniel Varon, Maria T. Greig, Peggy Roberts, Marilyn Albert, Chiadi Onyike, Daniel D’Agostino, Stephanie Kielb, James E. Galvin, Dana M. Pogorelec, Brittany Cerbone, Christina A. Michel, Henry Rusinek, Mony J. de Leon, Lidia Glodzik, Susan De Santi, P. Murali Doraiswamy, Jeffrey R. Petrella, Terence Z. Wong, Christopher M. Clark, Steven E. Arnold, Jason H. Karlawish, David A. Wolk, Charles D. Smith, Gregory Jicha, Peter Hardy, Partha Sinha, Elizabeth Oates, Gary Conrad, Oscar L. Lopez, MaryAnn Oakley, Donna M. Simpson, Anton P. Porsteinsson, Bonnie S. Goldstein, Kim Martin, Kelly M. Makino, M. Saleem Ismail, Connie Brand, Ruth A. Mulnard, Gaby Thai, Catherine McAdams-Ortiz, Kyle Womack, Dana Mathews, Mary Quiceno, Ramon Diaz-Arrastia, Richard King, Myron Weiner, Kristen Martin Cook, Michael Devous, Allan I. Levey, James J. Lah, Janet S. Cellar, Jeffrey M. Burns, Heather S. Anderson, Russell H. Swerdlow, Liana Apostolova, Kathleen Tingus, Ellen Woo, Daniel H. S. Silverman, Po H. Lu, George Bartzokis, Neill R. Graff-Radford, Francine Parfitt, Tracy Kendall, Heather Johnson, Martin R. Farlow, Ann Marie Hake, Brandy R. Matthews, Scott Herring, Cynthia Hunt, Christopher H. van Dyck, Richard E. Carson, Martha G. MacAvoy, Howard Chertkow, Howard Bergman, Chris Hosein, Sandra Black, Bojana Stefanovic, Curtis Caldwell, Ging-Yuek Robin Hsiung, Howard Feldman, Benita Mudge, Michele Assaly, Andrew Kertesz, John Rogers, Charles Bernick, Donna Munic, Diana Kerwin, Marek Marsel Mesulam, Kristine Lipowski, Chuang-Kuo Wu, Nancy Johnson, Carl Sadowsky, Walter Martinez, Teresa Villena, Raymond Scott Turner, Kathleen Johnson, Brigid Reynolds, Reisa A. Sperling, Keith A. Johnson, Gad Marshall, Meghan Frey, Jerome Yesavage, Joy L. Taylor, Barton Lane, Allyson Rosen, Jared Tinklenberg, Marwan N. Sabbagh, Christine M. Belden, Sandra A. Jacobson, Sherye A. Sirrel, Neil Kowall, Ronald Killiany, Andrew E. Budson, Alexander Norbash, Patricia Lynn Johnson, Thomas O. Obisesan, Saba Wolday, Joanne Allard, Alan Lerner, Paula Ogrocki, Leon Hudson, Evan Fletcher, Owen Carmichael, John Olichney, Smita Kittur, Michael Borrie, T.-Y. Lee, Rob Bartha, Sterling Johnson, Sanjay Asthana, Cynthia M. Carlsson, Steven G. Potkin, Adrian Preda, Dana Nguyen, Pierre Tariot, Stephanie Reeder, Vernice Bates, Horacio Capote, Michelle Rainka, Douglas W. Scharre, Maria Kataki, Anahita Adeli, Earl A. Zimmerman, Dzintra Celmins, Alice D. Brown, Godfrey D. Pearlson, Karen Blank, Karen Anderson, Robert B. Santulli, Tamar J. Kitzmiller, Eben S. Schwartz, Kaycee M. Sink, Jeff D. Williamson, Pradeep Garg, Franklin Watkins, Brian R. Ott, Henry Querfurth, Geoffrey Tremont, Stephen Salloway, Paul Malloy, Stephen Correia, Howard J. Rosen, Bruce L. Miller, Jacobo Mintzer, Kenneth Spicer, David Bachman, Elizabether Finger, Stephen Pasternak, Irina Rachinsky, Dick Drost, Nunzio Pomara, Raymundo Hernando, Antero Sarrael, Susan K. Schultz, Laura L. Boles Ponto, Hyungsub Shim, Karen Ekstam Smith, Norman Relkin, Gloria Chiang, Lisa Ravdin, Amanda Smith, Kristin Fargher, Balebail Ashok Raj, David A. Wolk, Ilya M. Nasrallah

**Affiliations:** 1 Department of Radiology, Perelman School of Medicine, University of Pennsylvania, Philadelphia, PA USA; 2 Department of Bioengineering, School of Engineering and Applied Sciences, University of Pennsylvania, Philadelphia, PA USA; 3 Department of Neurology, Perelman School of Medicine, University of Pennsylvania, Philadelphia, PA USA; 4 Alzheimer’s Disease Research Center, Perelman School of Medicine, University of Pennsylvania, Philadelphia, PA USA; 5 Department of Biostatistics, Epidemiology and Informatics, Perelman School of Medicine, University of Pennsylvania, Philadelphia, PA USA; 6 University of California San Francisco, San Francisco, CA USA; 7 University of California San Diego, San Diego, CA USA; 8 Mayo Clinic, Rochester, NY USA; 9 University of California Berkeley, Berkeley, CA USA; 10 University of Pennsylvania, Philadelphia, PA USA; 11 University of Southern California, Los Angeles, CA USA; 12 University of California Davis, Davis, CA USA; 13 Brigham and Women’s Hospital, Harvard Medical School, Boston, MA USA; 14 Indiana University, Bloomington, IND USA; 15 Washington University St. Louis, St. Louis, MO USA; 16 Janssen Alzheimer Immunotherapy, South San Francisco, CA USA; 17 University of Washington, Seattle, WA USA; 18 University of London, London, UK; 19 University of Michigan, Ann Arbor, MI USA; 20 University of Utah, Salt Lake City, UT USA; 21 Banner Alzheimer’s Institute, Phoenix, AZ USA; 22 University of Pittsburgh, Pittsburgh, PA USA; 23 University of California, Los Angeles, CA USA; 24 Khachaturian, Radebaugh & Associates, Inc and Alzheimer’s Association’s Ronald and Nancy Reagan’s Research Institute, Chicago, IL USA; 25 General Electric, Boston, MA USA; 26 Brown University, Providence, RI USA; 27 National Institute on Aging/National Institutes of Health, Bethesda, MD USA; 28 Oregon Health and Science University, Portland, OR USA; 29 Baylor College of Medicine, Houston, TX USA; 30 Columbia University Medical Center, New York, NY USA; 31 University of Alabama Birmingham, Birmingham, MO USA; 32 Mount Sinai School of Medicine, New York, NY USA; 33 Rush University Medical Center, Chicago, IL USA; 34 Wien Center, Vienna, Austria; 35 Johns Hopkins University, Baltimore, MD USA; 36 New York University, New York, NY USA; 37 Duke University Medical Center, Durham, NC USA; 38 Massachusetts General Hospital, Boston, MA USA; 39 University of Kentucky, Lexington, KY USA; 40 University of Rochester Medical Center, Rochester, NY USA; 41 University of California, Irvine, CA USA; 42 University of Texas Southwestern Medical School, Dallas, TX USA; 43 Emory University, Atlanta, GA USA; 44 University of Kansas, Medical Center, Lawrence, KS USA; 45 Mayo Clinic, Jacksonville, FL USA; 46 Yale University School of Medicine, New Haven, CT USA; 47 McGill University, Montreal Jewish General Hospital, Montreal, WI USA; 48 Sunnybrook Health Sciences, Toronto, ON Canada; 49 University of British Columbia Clinic for AD and Related Disorders, British Columbia, BC Canada; 50 Cognitive Neurology St. Joseph’s, Toronto, ON Canada; 51 St. Joseph’s Health Care, Toronto, ON Canada; 52 Cleveland Clinic Lou Ruvo Center for Brain Health, Las Vegas, NV USA; 53 Northwestern University, Evanston, IL USA; 54 Premiere Research Institute Palm Beach Neurology, West Palm Beach, FL USA; 55 Georgetown University Medical Center, Washington, DC USA; 56 Stanford University, Santa Clara County, CA USA; 57 Banner Sun Health Research Institute, Sun City, AZ USA; 58 Boston University, Boston, MA USA; 59 Howard University, Washington, DC USA; 60 Case Western Reserve University, Cleveland, OH USA; 61 University of California, Davis, Sacramento, CA USA; 62 Neurological Care of CNY, New York, NY USA; 63 Parkwood Hospital, Parkwood, CA USA; 64 University of Wisconsin, Madison, WI USA; 65 University of California, Irvine BIC, Irvine, CA USA; 66 Dent Neurologic Institute, Amherst, MA USA; 67 Ohio State University, Columbus, OH USA; 68 Albany Medical College, Albany, NY USA; 69 Hartford Hospital, Olin Neuropsychiatry Research Center, Hartford, CT USA; 70 Dartmouth Hitchcock Medical Center, Albany, NY USA; 71 Wake Forest University Health Sciences, Winston-Salem, NC USA; 72 Rhode Island Hospital, Providence, RI USA; 73 Butler Hospital, Providence, RI USA; 74 Medical University South Carolina, Charleston, SC USA; 75 Nathan Kline Institute, Orangeburg, SC USA; 76 University of Iowa College of Medicine, Iowa City, IA USA; 77 Cornell University, Ithaca, NY USA; 78 University of South Florida: USF Health Byrd Alzheimer’s Institute, Tampa, FL USA

**Keywords:** Alzheimer's disease, Prognostic markers, Neurodegeneration

## Abstract

Alzheimer’s disease (AD) is defined by amyloid (A) and tau (T) pathologies, with T better correlated to neurodegeneration (N). However, T and N have complex regional relationships in part related to non-AD factors that influence N. With machine learning, we assessed heterogeneity in ^18^F-flortaucipir vs. ^18^F-fluorodeoxyglucose positron emission tomography as markers of T and neuronal hypometabolism (N_M_) in 289 symptomatic patients from the Alzheimer’s Disease Neuroimaging Initiative. We identified six T/N_M_ clusters with differing limbic and cortical patterns. The canonical group was defined as the T/N_M_ pattern with lowest regression residuals. Groups resilient to T had less hypometabolism than expected relative to T and displayed better cognition than the canonical group. Groups susceptible to T had more hypometabolism than expected given T and exhibited worse cognitive decline, with imaging and clinical measures concordant with non-AD copathologies. Together, T/N_M_ mismatch reveals distinct imaging signatures with pathobiological and prognostic implications for AD.

## Abstract (web summary)

In Alzheimer’s disease (AD) tau and neurodegeneration have complex regional relationships. Here, the authors show neuronal hypometabolism discordant with tau burden defines functional resilience or susceptibility to Alzheimer’s pathology via limbic/cortical axes. Susceptible groups have faster cognitive decline and evidence of non-Alzheimer’s pathologies.

## Introduction

Alzheimer’s disease (AD) causes cognitive impairment with substantial between-patient variability in clinical presentation as well as the burden and distribution of pathology^1–3^. This clinicopathologic heterogeneity is both a challenge and opportunity for systematic, biomarker-based studies to refine our understanding of AD biology, diagnosis and management. AD hallmark pathologies begin with accumulation of amyloid (A) plaques, followed by deposition of tau (T) tangles and subsequent neuronal injury/neurodegeneration (N)^3^. A and T aggregates are bound by specialized radiotracers for in vivo positron emission tomography (PET) imaging (such as ^18^F-Flortaucipir for T tangles). N may be assessed via neuronal hypometabolism (N_M_) with ^18^F-fluorodeoxyglucose (^18^F-FDG) PET or structural atrophy (N_S_) with magnetic resonance imaging (MRI). Additional polypathologies contribute to clinical progression in AD, including vascular and inflammatory etiologies, α-synucleinopathy and TAR DNA-binding protein-43 (TDP-43) diseases, many of which do not currently have specific in vivo measures^3,4^.

To address this complexity and provide a biological, rather than clinical, definition of AD, the National Institute on Aging and Alzheimer’s Association proposed the ATN research framework^3^. These criteria designate the global presence (+) or absence (−) of three AD dimensions: A, T and N. Patients with A+ status are included in the Alzheimer’s continuum while a research diagnosis of AD necessitates both A+ and T+, consistent with the definition of AD neuropathologic change on autopsy. This model consolidates various pathological interactions in the Alzheimer’s continuum to classify heterogeneous groups by a panel of dichotomized biomarkers. Such categorical approach has already shed light on differential rates of memory decline^5,6^ and clinical risks/outcomes^7,8^ in patients with certain ATN combinations.

Neurodegeneration in AD is largely thought to be driven by T neurofibrillary tangles^9,10^ and much work has supported a strong spatial, quantitative link between measures of T and N_M_^11–13^. However, both T and N show variability in patterns across the brain and between individuals, and this T/N relationship is not a complete one-to-one mapping^14,15^. Compared to a typical relationship between deposition of neurofibrillary tangles and neuronal hypometabolism (N_M_ ~ T), a relative decoupling of T and N_M_ may arise when patients have less N than expected given their T level (N_M_ < T as metabolic resilience to T), or greater N than expected given their T (N_M_ > T as susceptibility). Quantification of relative T/N_M_ mismatch may capture resilience and vulnerability in neuronal metabolic responses to T, perhaps linked to non-AD pathophysiology not currently operationalized within ATN criteria.

**Fig. 1:**
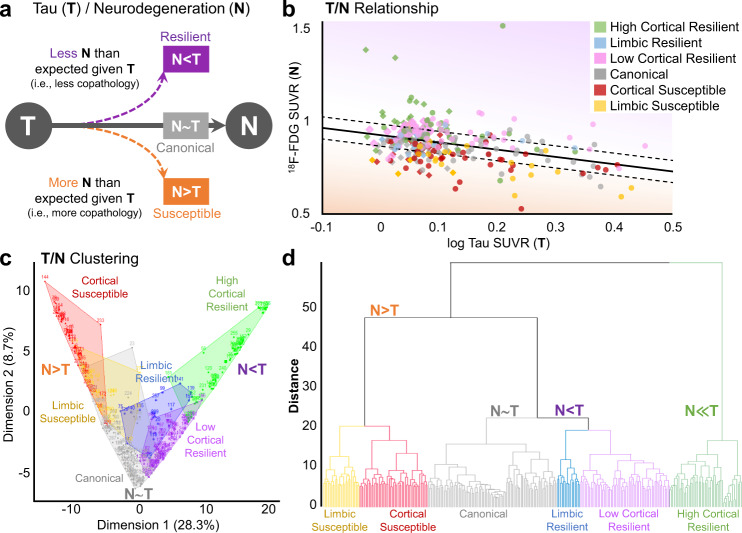
T/N_M_ mismatch by clustering of the whole cohort. **a** Schematic of proposed relationship between tau (T) and neurodegeneration (N) by neuronal hypometabolism (N_M_). **b** Regression model of ^18^F-FDG vs. log tau SUVR in the inferior temporal gyrus, a typical tau staging region in AD. Solid line represents the model, with dashed lines denoting standard deviation-based thresholds (*n* = 289 participants). N_M_ < T denotes points above the line and N_M_ > T depicts points below the line. Circles are A+. Diamonds are A− participants. Source data are provided as a Source Data file. Consistent clustering by T/N_M_ mismatch of all regional and patient residuals is visually demonstrated by (**c**) principal component analysis and (**d**) dendrogram.

Here, we developed a machine learning-based clustering method to identify mismatch between T and N_M_ using symptomatic patients from the Alzheimer’s Disease Neuroimaging Initiative (ADNI) cohort. We posited that mismatch analyses from PET markers of T and N_M_ would reveal imaging signatures of patient groups including a typical or canonical T/N_M_ relationship as well as unique patterns of resilience and susceptibility to T. We hypothesized for a given level of T, susceptible patients with greater than expected N_M_ have worse cognitive decline compared to participants with canonical T/N_M_ relationships, potentially due to more concomitant non-AD pathologies than the canonical group (Fig. 1a). Given that AD autopsy studies reveal widespread prevalence of non-AD copathology^16^, we predicted that some of the dissociation between T and N_M_ is attributable to a spectrum of mixed disease burden. The N_M_ > T scenario may encompass patients with metabolic vulnerability to T along with the presence of non-AD copathologies such as α-synuclein and TDP-43 that contribute to N_M_ independently of T and at levels greater than the canonical group. Moreover, we expected that the canonical group likely has some intermediate amount of mixed disease, while resilient groups may have less copathology and slower cognitive decline

To this end, we evaluated T/N_M_ mismatch and its relation to clinical features, cognitive progression and supportive evidence for copathologies. Since non-AD pathologies and risk factors are expected to be present in both A+ or A− individuals, we performed *post hoc* analyses in the whole cohort and A+ or A− groups. Our findings were replicated with a cohort of cognitively normal older adults in the Harvard Aging Brain Study (HABS). Overall, we demonstrate the utility of T/N_M_ mismatch in modeling AD heterogeneity, predicting progression and providing pathophysiological insight for cognitive impairment.

## Results

### T/N_M_ mismatch defines groups by regional residual patterns

We measured the relationship between T and N_M_ (Fig. 1a) by regressions of ^18^F-FDG vs. tau standardized uptake value ratios (SUVRs) for each region-of-interest (ROI) and individual. Within our ADNI cohort (*n* = 289, Supplementary Table 1), clustering on T/N_M_ regression residuals resulted in six groups with sizes ranging from 16 to 89 members. These groups were labeled based on the relative spatial pattern of metabolic resilience or vulnerability to T, which we describe below. As an example, group identity (the cluster to which a participant belongs) was mapped onto graphs for regions such as inferior temporal gyrus (Fig. 1b). This ROI is involved in early symptomatic stages of AD progression and is a representative of between-group differences in T/N_M_ relations^5,17^. Similar T/N_M_ relations were seen in residual heatmaps across all ROIs and patients (Supplementary Fig. 1). Next, we assessed the consistency of our clustering across different visualization methods. A principal component analysis (PCA) (Fig. 1c) and *t*-distributed stochastic neighbor embedding (*t*-SNE) method (Supplementary Fig. 1) map the 104 ROI dimensions onto two axes and both corroborated the between-group separation of clusters. A dendrogram visualized the within-group similarity across clustered patients (Fig. 1d). Therefore, the consistency of these groups across several dimensionality reduction methods substantiates this clustering approach.

**Fig. 2:**
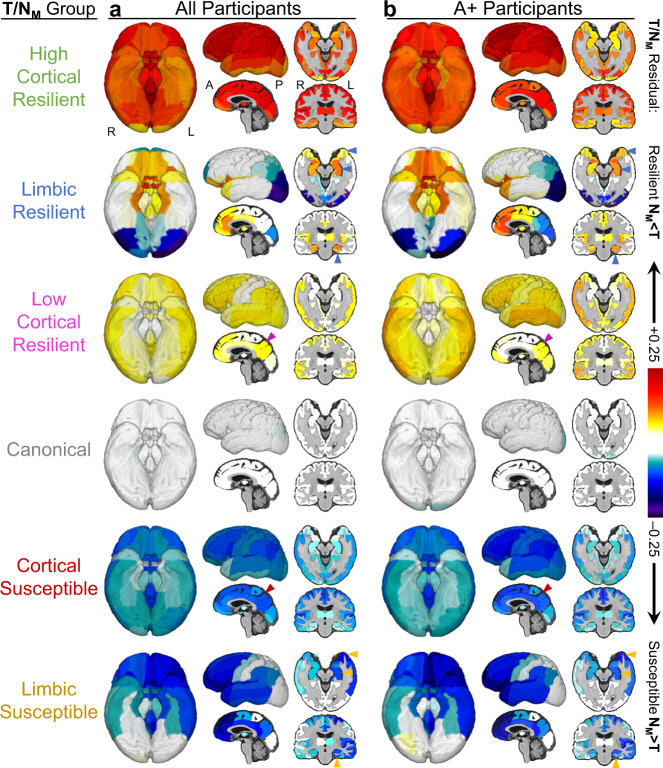
Brain maps visualize T/N_M_ mismatch relationships and spatial patterns. Three- and two-dimensional renderings of mean T/N_M_ relation regional residuals are shown for **a** all participants and **b** A+ patients. Compared to the canonical (N_M_ ~ T) group, resilient (N_M_ < T) and susceptible (N_M_ > T) groups have limbic vs. cortical involvement (arrowheads). Color scale represents the mean T/N_M_ residual (in ^18^F-FDG SUVR). R right, L left, A anterior, P posterior.

There was no significant between-group difference in A status. Despite this lack of statistically significant difference, some groups appeared more enriched in A+ individuals, so we covaried by A status, as well as for sex, age, education level and T burden in the inferior temporal gyrus in subsequent omnibus and between-group analyses. There were between-group differences, including significant differences in sex and education, across all participants (Table 1) and specifically among A+ (Table 2) or A− patients (Supplementary Table 2). There were no significant differences in age. The groups had similar average tau SUVRs across all regions; the distribution of individuals with regional T patterns that correspond to AD Braak stages were also similar across groups for all participants (Fig. 2). Hence, these groups likely do not depict distinct stages of AD progression but instead appear to represent unique spatial patterns of the relationship between T pathology and its functional consequences.

**Table 1:** T/N_M_ mismatch clustering across all participants.

Group	A−/A+	MCI/Dem	F/M	Age (y)	Educ (y)	Cognition			Cortical thickness (mm)
						ADAS-Cog	CDR-SOB	MMSE	
High cortical resilient	27/23	45/5	24/26	74.1 (7.7)	16.3 (2.6)	19.6 (7.3)	1.8 (1.6)	27.8 (2.6)^+^	2.09 (0.36)^+^
Limbic resilient	9/7	14/2	7/9	75.4 (10.2)	16.1 (2.6)	20.0 (7.6)	2.0 (1.2)	27.1 (3.8)	2.00 (0.33)
Low cortical resilient	26/36	57/5	32/30	71.6 (7.9)	15.6 (2.4)	18.8 (5.8)**	1.7 (1.5)^+^	27.7 (2.1)**	2.10 (0.37)*
Canonical	40/49	60/29	33/56	74.9 (7.8)	16.5 (2.6)	22.9 (8.8)	2.5 (2.2)	26.7 (3.0)	1.87 (0.51)
Cortical susceptible	16/31	26/21	14/33	74.8 (13.4)	15.4 (3.3)	26.0 (8.4)	3.5 (2.5)	25.1 (3.4)^+^	1.83 (0.37)
Limbic susceptible	7/18	12/13	14/11	78.9 (6.1)	16.2 (2.4)	25.6 (9.6)	3.2 (2.4)	25.3 (4.0)	1.65 (0.40)
Group *p* val			0.03	0.26	0.21	0.001	0.01	0.03	0.02

Amyloid (A) status (A+/A−), diagnosis (MCI/dementia) and sex (F/M) are in frequencies. Mean (standard deviation) values are shown for age/education (years), AD Assessment Scale-Cognitive (ADAS-Cog, higher is worse), Clinical Dementia Rating sum of boxes (CDR-SOB, higher is worse), Mini-Mental Status Exam (MMSE, lower is worse) and global cortical thickness (mm). The last row depicts group difference *p* values by likelihood ratio tests after adjusting for covariates. Significant differences in pairwise comparisons between a non-canonical and canonical group with covariate adjustment are annotated. For pairwise comparisons, * denotes *p* < 0.05 after multiple tests adjustment, ** denotes *p* < 0.005 after multiple tests adjustment and ^+^ denotes *p* < 0.05 before multiple tests adjustment. Covariates include sex, age, education, A status and inferior temporal gyrus tau SUVR. Sample sizes and *p* values are listed in Supplementary Data 1.

**Table 2:** T/N_M_ mismatch clustering across A+ patients.

Group	MCI/Dem	F/M	Age (y)	Educ (y)	Cognition			Cortical thickness (mm)
					ADAS-Cog	CDR-SOB	MMSE	
High cortical resilient	19/4	11/12	74.5 (6.8)	16.5 (2.5)	22.5 (8.6)	2.0 (1.8)	27.1 (3.4)	2.05 (0.25)
Limbic resilient	5/2	4/3	70.4 (11.5)	15.6 (2.3)	23.5 (9.0)	2.4 (1.2)	24.7 (4.7)	1.95 (0.42)
Low cortical resilient	31/5	20/16	73.4 (7.4)	15.0 (2.1)	20.7 (5.8)**	2.0 (1.8)^+^	27.1 (2.2)**	2.08 (0.22)**
Canonical	26/23	19/30	75.6 (7.4)	16.6 (3.6)	26.5 (8.4)	3.0 (2.2)	25.6 (3.1)	1.77 (0.57)
Cortical susceptible	14/17	10/21	74.3 (15.6)	15.6 (3.1)	28.4 (8.4)	4.0 (2.5)^+^	24.2 (3.6)	1.78 (0.41)
Limbic susceptible	7/11	11/7	78.8 (5.3)	15.7 (2.2)	28.0 (9.6)	3.3 (2.5)	24.7 (4.4)	1.64 (0.44)
Group *p* val		0.03	0.36	0.01	<0.0001	0.01	<0.05	0.005

Diagnosis (MCI/dementia) and sex (F/M) are in frequencies. Mean (standard deviation) values are shown for age/education (years), ADAS-Cog, CDR-SOB, MMSE, and global cortical thickness (mm). The last row depicts group difference *p* values by likelihood ratio tests after adjusting for covariates. Significant differences in pairwise comparisons between a non-canonical and canonical group with covariate adjustment are annotated. For pairwise comparisons, ** denotes *p* < 0.005 after multiple tests adjustment and ^+^ denotes *p* < 0.05 before multiple tests adjustment. Covariates include sex, age, education and inferior temporal gyrus tau SUVR. Sample sizes and *p* values are listed in Supplementary Data 1.

Herein, we characterize our six T/N_M_ mismatch groups. The largest group of individuals (89/289) were found close to the regression line across most regions, with the smallest residuals. This canonical group defines the condition where relative N_M_ was statistically commensurate to the level of T (N_M_ ~ T). The other five groups were compared to the canonical group by T/N_M_ residuals in three- and two-dimensional regional maps across all participants (Fig. 2a), visualizing regions where N_M_ is greater or less than what is observed in the canonical group given the T level. Groups derived from clustering all participants showed distinct neuroanatomical patterns; these patterns were similar across subcohorts of A+ patients (Fig. 2b) and A− patients (Supplementary Fig. 3).

There were 3 groups with less N relative to their T level compared to the canonical group (positive residuals), thus classified as resilient to T (Fig. 2). The resilient groups had relative differences in spatial patterns of T/N_M_ mismatch corresponding to prominent regions either throughout the cerebral cortex, termed the cortical resilient groups, or limbic areas, termed the limbic resilient group. Cortical resilient patterns stratified into two groups based on either high or low magnitude residuals. The high cortical resilient group (50/289) had higher T/N_M_ residuals across most cortical and limbic ROIs compared to the canonical group and was the first group to split in clustering (Fig. 1d). The low cortical resilient group (62/289) had positive residuals throughout the cortex compared to the canonical or limbic resilient groups. While both cortical resilient groups had similar T levels (Supplementary Fig. 2), the high cortical resilient group had greater T/N_M_ residuals (Fig. 2). Both high and low cortical resilient groups had similar distributions of positive residuals but the low cortical resilient group had lower magnitude residuals, especially in limbic structures. The limbic resilient group (16/289) had high positive T/N_M_ residuals localized to the medial temporal lobe (MTL), anterior temporal and orbitofrontal regions compared to the canonical or other resilient groups, while other cortical regions had lower residuals here relative to the canonical group.

Two groups had worse N than typical for their level of T (negative residuals) and were considered susceptible to T (Fig. 2). These groups also had a relative predilection forspatial patterns involving predominantly cortical or limbic regions, though these regional distributions were less distinct compared to those in the resilient groups. The cortical susceptible group (47/289) had lower residuals generally in cortical regions, with lesser extent in limbic regions than other groups. The limbic susceptible group (25/289) had a pattern of low residuals in primarily limbic and anterior frontotemporal areas.

### T/N_M_ groups have differences in N but not T markers

**Fig. 3:**
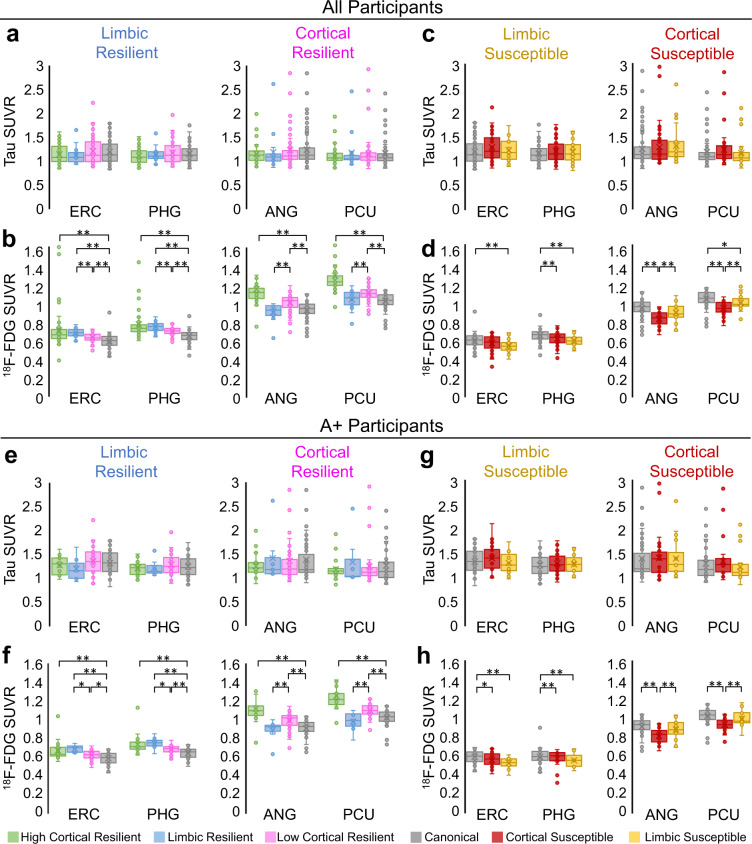
T/N_M_ mismatch depicts differences in regional ^18^F-FDG but not tau SUVR. PET uptake is graphed in **a**–**d** all participants and **e**–**h** A+ patients. Resilient groups had (**a**, **e**) similar tau across regions and (**b**, **f**) unique ^18^F-FDG SUVRs in limbic and cortical regions. Susceptible groups had (**c**, **g**) similar tau across regions and (**d**, **h**) unique ^18^F-FDG SUVRs in limbic and cortical regions. Box plots show data points as dots, mean as an X symbol, median as the middle box line, first quartile (Q1) and third quartiles (Q3) as box edges (denoting the interquartile range, IQR), whiskers as the minimum/maximum points and outliers based on thresholds <Q1 − 1.5(IQR) or >Q3 + 1.5(IQR). Significant differences in pairwise comparisons with the canonical group by two-tailed likelihood ratio tests after covariate and multiple test (Benjamini–Hochberg) adjustment are denoted as *p < 0.05, **p < 0.005. Covariates include sex, age, education, A status and T level. Sample sizes and *p* values are provided in Supplementary Data 1. Source data are provided as a Source Data file. Abbreviations for limbic regions [entorhinal cortex (ERC), parahippocampal gyrus (PHG)] and cortical regions [angular gyrus (ANG), precuneus (PCU)].

We evaluated whether clustering in T/N_M_ residuals was generally driven by either tau or ^18^F-FDG SUVR. Notably, our groups did not significantly vary by T burden (Supplementary Fig. 2), indicating that residual-based clustering was more influenced by between-group^18^F-FDG SUVR differences, even after covarying for sex, age, education, A status and T level. Among resilient groups, T/N_M_ residual patterns (Fig. 2) were not linked to regional differences in tau SUVR (Fig. 3a), but rather ^18^F-FDG SUVR (Fig. 3b). The high cortical resilient group had significantly higher covariate-adjusted^18^F-FDG SUVR across several representative regions compared to the canonical and other resilient groups (*p*’s < 0.005). Significant differences between covariate-adjusted^18^F-FDG SUVR in the limbic and low cortical resilient groups matched the group differences in T/N_M_ residuals. Compared to the canonical group, the limbic resilient group had significantly higher ^18^F-FDG SUVR in MTL structures while the low cortical resilient group had elevated ^18^F-FDG SUVR throughout the cortex (*p*’s < 0.005). Likewise, across susceptible groups, there were no regional differences in tau SUVR (Fig. 3c), but instead ^18^F-FDG SUVR (Fig. 3d). Compared to the canonical group, the limbic susceptible group had lower ^18^F-FDG SUVR in limbic areas while the cortical susceptible group had worse ^18^F-FDG SUVR in other cortical regions (*p*’s < 0.005). Regional resilience and susceptibility patterns across the cohort (Fig. 3a–d) were replicated in subgroups of A+ patients (Fig. 3e–h) and A− patients (Supplementary Fig. 4).

Additionally, mean cortical thickness differed among groups (Tables 1 and 2). In the whole cohort, thickness was greater in the low cortical resilient (2.10 mm, *p* = 0.01, unadjusted) and high cortical resilient group (2.09 mm, *p* = 0.04, unadjusted) compared to the canonical group (1.87 mm).

### T/N_M_ clustering shows consistency across internal and external validation

We aimed to internally validate our clustering approach within those participants demonstrating AD pathologic change. We performed clustering on A+ participants only, who overall also demonstrate higher T burden (since 87% of A+ participants were T+). Indeed, groups generated from A+ participants alone resembled groups formed from clustering all participants, in overall patterns and group identity (Supplementary Fig. 5). Then, we compared the robustness of clustering on subsets of 150 randomly selected participants over ten folds. Clustering was stable across folds (Supplementary Fig. 6). About 90% of participants had a match between their original group identity and the group identity endorsed by a majority of folds, while 9% of participants had group identity shift in the same residual direction (such as between high and low cortical resilience). These experiments demonstrate the robustness of our clustering.

Because clustering was similar across A+ and A− cognitively impaired ADNI participants, next we corroborated clustering in the external HABS cohort of cognitively normal older adults with lower levels of A and T pathology (Supplementary Tables 3 and 4). Six T/N_M_ groups were generated from the whole HABS cohort, demonstrating similar regional patterns to those found in symptomatic ADNI participants: canonical, high and low cortical resilience, limbic resilience, cortical susceptibility and limbic-predominant susceptibility (Supplementary Fig. 7). Thus, patterns of T/N_M_ dissociation similar to those in symptomatic AD may be shared across settings of preclinical AD and cognitive aging, where resilience factors or non-AD pathologies may influence ^18^F-FDG metabolism at low or intermediate levels of T.

### T/N_M_ groups exhibit different cognitive trajectories

We hypothesized that relative hypometabolism for a given level of T may be associated with differences in cross-sectional and longitudinal cognitive measures. Although T and N markers both strongly associate with cognitive impairment, we predicted that susceptible participants may have additional copathologies contributing to N and leading to greater cognitive decline than predicted by T. We found significant cross-sectional group differences across the cohort for various cognitive tests at the time of ^18^F-FDG scan even after controlling for covariates such as sex, age, education, baseline cognition, A status and T level (Table 1). In absolute terms, the canonical group had mid-range impaired ADAS-Cog (22.9), while resilient groups had lower, better scores (19.6, 20.0, 18.8) and susceptible groups had higher, worse scores (25.6, 26.0). These results were replicated with additional global cognitive measures (Mini-Mental Status Exam (MMSE) and Clinical Dementia Rating sum of boxes (CDR-SOB)). For cross-sectional covariate-adjusted pairwise comparisons, significant differences were noted between the canonical and low cortical resilient groups on the ADAS-Cog (*p* = 0.0003) and MMSE (*p* = 0.002). Such differences were also seen in the A+ cohort (Table 2).

**Fig. 4:**
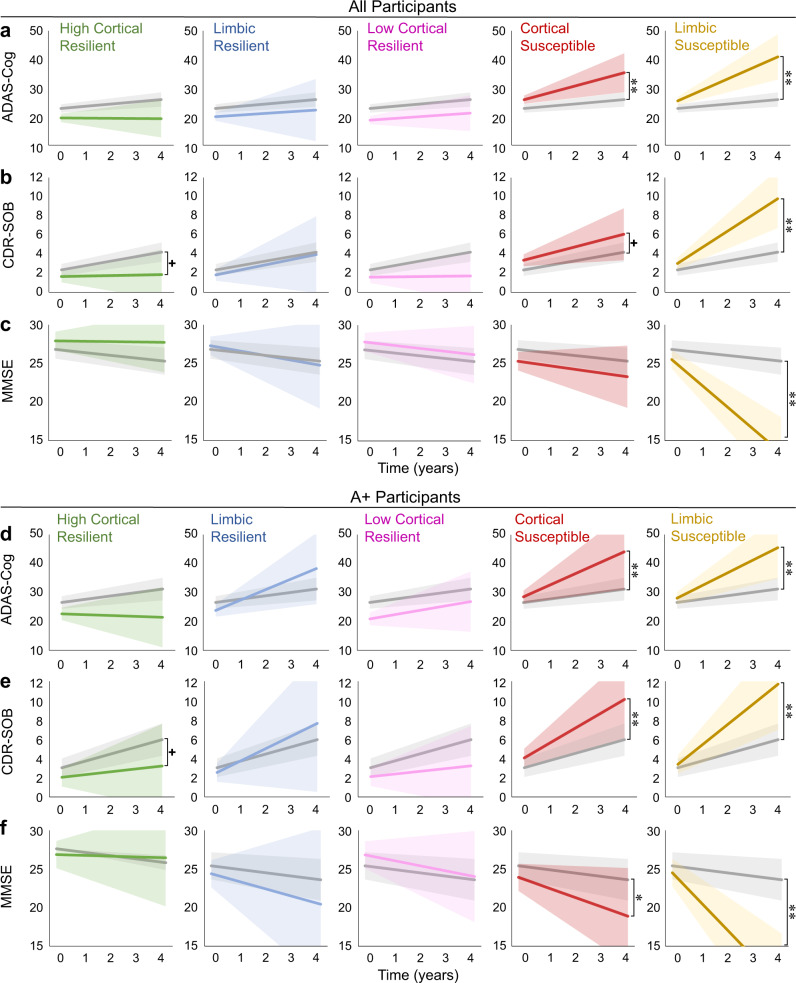
Differential cognitive decline based on T/N_M_ mismatch. Longitudinal cognitive trajectories differ by group identities among (**a**–**c**) all participants and (**d**–**f**) A+ participants. Decline rates are shown for (**a**, **d**) AD Assessment Scale Cognitive 13 item (ADAS-Cog, higher score is worse), (**b**, **e**) Clinical Dementia Rating Sum of Boxes (CDR-SOB, higher is worse) and (**c**, **f**) Mini-Mental Status Exam (MMSE, lower is worse) by linear mixed effects models with amyloid status, baseline score, education, sex, age and T level as covariates. Lines show the mixed effect model and error bands show ±1 propagated standard error. Significant differences in pairwise comparisons of cognitive decline between a non-canonical and canonical group by linear mixed effects analysis with multiple test (Benjamini–Hochberg) adjustment are denoted as *p < 0.05, **p < 0.005. ^+^denotes *p* < 0.05 before multiple test adjustment. Sample sizes and *p* values are provided in Supplementary Data 1. Source data are provided as a Source Data file.

Then, we compared longitudinal cognitive trajectories by linear mixed effects models with covariates (Fig. 4 and Supplementary Table 5). Across groups, the canonical group had mid-range decline on ADAS-Cog (+0.8 points/year) (Fig. 4a). The resilient groups (high cortical, limbic, low cortical) had the slowest progression on ADAS-Cog (–0.07, +0.6, +0.6 points/year, respectively). Though ADAS-Cog slopes in resilient groups did not significantly differ from the canonical group, the high cortical resilient group showed less decline on CDR-SOB than the canonical group (*p* = 0.04, uncorrected). In contrast, there was significantly steeper decline on ADAS-Cog in the cortical susceptible (+2.4 points/year, *p* = 0.002) and limbic susceptible groups (+3.9 points/year, *p* < 0.0005) than the canonical group (Fig. 4a). Significant differences between canonical and susceptible trajectories were also found for CDR-SOB and MMSE (Fig. 4b,c). Among A+ participants (Fig. 4d–f) and A− patients only (Supplementary Fig. 8), between-group differences in cognitive progression rates were comparable to the whole cohort.

Akin to cognitively impaired ADNI participants, cognitively normal HABS participants had a significant group difference in cross-sectional MMSE (*p* = 0.008) (Supplementary Table 4). On MMSE, groups corresponding to T/N_M_ susceptibility had significantly lower baseline scores. Together, our data suggests that the decoupling of T and N_M_ may relate to factors affecting cognitive outcomes in both symptomatic and asymptomatic individuals across the distribution of T level.

### Exploratory analysis of copathology factors driving T/N_M_ mismatch

**Fig. 5:**
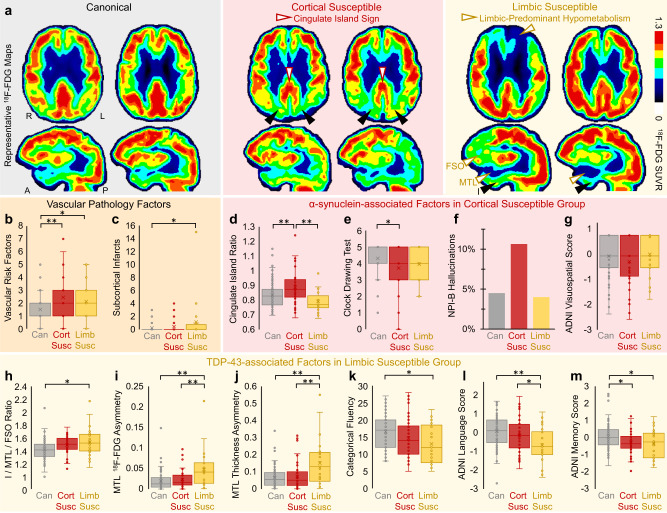
Exploratory analysis of vascular, α-synuclein (Lewy body) and TDP-43 copathology features in T/N_M_ susceptible groups. **a** Representative ^18^F-FDG SUVR images from six patients. Susceptible patients shown here have imaging findings consistent with copathology (sagittal views are slices through the right hemisphere). Vascular pathology features in susceptible groups included greater (**b**) vascular risk factors and (**c**) subcortical infarcts. The cortical susceptible group had participants with (**a**) cingulate island sign, the sparing of posterior cingulate cortex (white arrowheads) relative to cuneus (black arrowheads) in representative ^18^F-FDG images, quantified by higher (**d**) cingulate island ratio across groups. The cortical susceptible group had (**e**) significantly worse clock drawing scores and trended toward greater (**f**) proportion of participants with hallucinations on the Neuropsychiatric Inventory (NPI) item B and worse (**g**) ADNI visuospatial *z*-scores than the other groups. The limbic susceptible group had participants with (**a**) medial temporal lobe (MTL) and frontal supraorbital (FSO) ^18^F-FDG hypometabolism (white arrowheads) relative to inferior temporal gyrus (I, black arrowheads) in representative ^18^F-FDG images, quantified by larger (**h**) I/MTL/FSO ^18^F-FDG ratio and worse MTL asymmetry in (**i**) ^18^F-FDG SUVR and (**j**) thickness. The limbic susceptible group had the significantly worse (**k**) categorical fluency, (**l**) language and (**m**) memory *z*-scores. Box plots show data points as dots, mean as an X symbol, median as the middle box line, first quartile (Q1) and third quartiles (Q3) as box edges (denoting the interquartile range, IQR), whiskers as the minimum/maximum points and outliers based on thresholds <Q1 − 1.5(IQR) or >Q3 + 1.5(IQR). Cognitive test comparisons included A status, education, sex and age as covariates. Significant differences in pairwise comparisons by two-tailed likelihood ratio tests are denoted by *p < 0.05, **p < 0.005. These results were not corrected for multiple comparisons due to their exploratory nature. Sample sizes and *p* values are provided in Supplementary Data 1. Source data are provided as a Source Data file.

Since susceptible groups had greater N than expected given their T and faster cognitive progression, we considered potential roles of copathology in driving advanced N (Fig. 5a). The cortical and limbic susceptible groups had significantly greater number of vascular clinical risk factors than the canonical group (*p* = 0.0003, *p* = 0.04, respectively) (Fig. 5b). The limbic susceptible group had significantly higher average subcortical infarct burden than the canonical group (*p* = 0.04) (Fig. 5c). White matter hyperintensity (WMH) volumes were higher in susceptible groups compared to the canonical group though such trends were not significant (Supplementary Fig. 9A). We also explored *APOE*, a gene harboring a common variant linked to dementia. APOE4 risk allele frequency was higher in the susceptible groups than other groups but not significantly different than the canonical group (Supplementary Fig. 9B).

Next, we studied how mixed proteinopathies may contribute to susceptible groups. While there are no definitive imaging or cognitive markers for the presence of α-synuclein or TDP-43, we assessed the consilience of several suggestive imaging and cognitive tests to provide some indication for what additional copathologies may be present in the setting of T/N_M_ mismatch.

We tested imaging and clinical markers of α-synuclein (Lewy body) pathology in the cortical susceptible group. A well-studied, potential indicator of Lewy Body Disease (LBD) is the cingulate island sign^18–20^, the relative sparing of ^18^F-FDG SUVR in the posterior cingulate cortex relative to precuneus and cuneus (Fig. 5a). There was significantly higher cingulate island ratio in the cortical susceptible group compared to the limbic susceptible and canonical groups (*p*’s < 0.005, Fig. 5d). Differences were also significant in A+ and A− cohorts. We also assessed cognitive features linked to LBD, such as visuospatial impairment^17^. Compared to the canonical group, the cortical susceptible group had significantly worse covariate-adjusted Clock Drawing scores (*p* = 0.03) and other visuospatial markers (Fig. 5e and Supplementary Fig. 9C) and trended toward higher proportion of patients with hallucinations on the Neuropsychiatric Inventory (NPI) and worse visuospatial *z*-scores (Fig. 5f, g). Thus, these imaging and cognitive results suggest potential α-synuclein pathology in the cortical susceptible group.

We analyzed the possibility of Limbic-predominant Age-related TDP-43 Encephalopathy (LATE) copathology in the limbic susceptible group given a pattern of severe anterior temporal/MTL hypometabolism relative to T (Figs. 2 and 5a). We utilized the I/MTL/FSO ratio, defined as worse MTL and frontal supraorbital (FSO) hypometabolism relative to inferior temporal gyrus (I). Higher I/MTL/FSO ratio signifies worse MTL hypometabolism and correlates to LATE in clinicopathologic studies^21,22^. The limbic susceptible group had significantly higher I/MTL/FSO ^18^F-FDG ratio relative to the canonical group (*p* = 0.01) (Fig. 5h). LATE commonly presents with asymmetric hippocampal sclerosis^23^, so we evaluated MTL asymmetry indices^24^ for ^18^F-FDG hypometabolism and atrophy. The limbic susceptible group had significantly higher MTL asymmetry in ^18^F-FDG SUVR and thickness (Fig. 5i, j) than canonical and cortical susceptible groups (*p*’s < 0.005). We further evaluated memory phenotypes linked to LATE^23^. Compared to the canonical group, the limbic susceptible group had worse semantic memory with significant covariate-adjusted differences in category fluency (*p* = 0.02) (Fig. 5k), Multilingual Naming Test (*p* = 0.008) (Supplementary Fig. 9D) and ADNI domain *z*-scores for language and memory (*p*’s < 0.05) (Fig. 5l, m). These imaging and cognitive profiles imply possible TDP-43 pathology in the limbic susceptible group.

Overall, these findings suggest that symptomatic susceptible groups had more copathology-related factors than the canonical group. Cognitively normal groups resembling T/N_M_ susceptibility patterns in the HABS cohort also demonstrate covariate-adjusted biomarker elevations consistent with greater non-AD pathology (Supplementary Fig. 10). When we evaluate the same biomarkers of copathology in the resilient groups, we generally observed less evidence of mixed pathology burden than the canonical group, particularly the cingulate island ratio as well as I/MTL/FSO ratio and MTL thickness asymmetry (Supplementary Fig. 11). Overall, non-AD pathology biomarkers convey higher burden in susceptible groups and lower burden in resilient groups compared to the canonical group, indicating that relative levels of mixed pathologies contribute to T/N_M_ mismatch and that canonical patients have some degree of copathology concordant with their commonality in autopsy series^16^.

## Discussion

We leveraged paired tau and ^18^F-FDG PET studies to assess the in vivo dissociation of T and N_M_ relationships in cognitively impaired individuals in the ADNI dataset. Clustering identified six groups of patients, including a canonical group that defines the expected relationship between T and N_M_ (N_M_ ~ T) and additional groups that were either more resilient (N_M_ < T) or susceptible (N_M_ > T) to T, defined by less or greater N_M_ than expected for a given level of T, respectively. We also clustered residuals from the T/N_M_ relationship across ten folds on random subsets of participants, and with A+ participants only, which did not impact overall clustering. Groups resembling our six T/N_M_ groups in symptomatic ADNI participants appeared in the asymptomatic HABS cohort, further validating the spatial patterns presented here.

Our T/N_M_ groups had significant differences in ^18^F-FDG and not tau SUVR at a group level (Fig. 3). This fact does not necessarily signify that T/N_M_ clustering was solely driven by ^18^F-FDG, but rather that clustering depends on variation in ^18^F-FDG relative to tau SUVR at an individual level. Certain participants can be identified with similar cortical ^18^F-FDG SUVR, but vastly different tau SUVR. For instance, a patient with high tau SUVR may have lower N_M_ (more metabolism) than expected given their level of T and may be placed in the low cortical resilient group, whereas a patient with low tau SUVR may have higher N_M_ (less metabolism) than expected given their T and may fall in the cortical susceptible group. Compared to clustering on just N_M_, T/N_M_ clustering enables regional and relative comparisons of N_M_ given a level of T and promotes the evaluation of factors beyond AD stage or pathology that may not be captured from N_M_ alone.

Relative to the canonical group, the resilient groups had better baseline cognitive scores whereas susceptible groups had faster cognitive decline over time. Metabolic and cognitive phenotypes in the T/N_M_ resilient and susceptible groups were shared across A+ and A− cohorts. The observations of impaired cognition and hypometabolism in A− susceptible groups strengthen the notion that factors influencing the T/N_M_ relationship in AD (like copathology) are also present in non-AD symptomatic patients. In other words, the A+ group may reflect AD plus additional factors, including copathologies, whereas the A− group may have these non-AD factors alone affecting this clustering. Our results support the use of T/N_M_ mismatch as a complement to direct measures of ATN biomarkers to study disorders of AD and non-AD pathology.

It is important to note that T/N_M_ groups also differed with regard to cortical atrophy (Tables 1 and 2). Since N_S_ and N_M_ are linked, it is reasonable to predict that T/N_S_ and T/N_M_ relationships are also associated. Indeed, clustering with T/N_S_ approaches yields groups with relative resilience or vulnerability to T^25^. The median regional correlation coefficient between T/N_S_ and T/N_M_ residuals was 0.29, suggesting that while T/N_S_ and T/N_M_ relationships are similar, they may provide some unique information. For example, metabolism may be more sensitive to Lewy body pathology than structure^18,19^. Likewise, metabolism may reflect aspects of functional reserve and synaptic activity not captured by structural markers while, alternatively, structure may be less affected by non-disease related functional variability than metabolism. Thus, it is likely the case that T/N_S_ and T/N_M_ mismatch each offer complementary, yet unique characterizations.

Intriguingly, both resilient and susceptible groups appeared to divide along patterns roughly involving either limbic or cortical regions. Several studies demonstrate similar spatial separation. For example, heterogeneity in either T or N alone has been examined by regional involvement and disease trajectories^15,26–28^. Here, we investigate the variability in relationships between T and N_M_ biomarkers with an integrative approach that evinces consistent patterns of neuroimaging and cognitive measures within the disentangling of N_M_ relative to T. To some extent, this dividing line between limbic vs. cortical involvement perhaps parallels the dissociable MTL networks described by^29–32^. This previous work supports the existence of the anterior temporal network, most akin to the limbic regions described here, and a posterior-medial network that largely conforms to the default mode network. Prior research has also suggested differential changes within these networks across the AD continuum^29,33,34^. Non-AD pathologies, such as TDP-43, might also split along this anterior-posterior axis^35^. Though we observed two cortical resilient groups (high and low), it is unclear what factors beyond metabolism distinguish high and low cortical resilience. These T/N_M_ differences were enough for these groups to strongly cluster separately since the high cortical resilient group was the first group to separate in terms of dendrogram distance (Fig. 1d). That said, these two groups may reflect a continuum of cortical resilience. Together, our findings may indicate differentially connected networks harbor not only dissociable vulnerabilities to accumulation of different pathologies, but also relative resiliencies to these pathological states.

We probed several factors that may influence the link between T and N_M_, including association of surrogate markers for three copathologies (vascular disease, α-synuclein and TDP-43). While our groups did not differ in mean tau SUVR burden or inferred Braak stage distribution (Fig. 3 and Supplementary Fig. 2), they did separate in terms of cognitive profiles, progression, and copathology-associated markers, suggesting that non-AD pathologies contribute to the dissociation of T and N_M_ and, thus, to the cognitive trajectory beyond Braak stage. In fact, longitudinal group differences were found even when covarying for baseline tau SUVR, which further suppresses effects of AD severity. Other aspects of resilience or vulnerability outside copathology also may influence outcomes beyond Braak staging. To this point, there was evidence of greater burden of vascular disease, a common copathology in AD^3^, in the susceptible patients, suggesting that the elevated levels of cerebrovascular disease compared to the canonical group are a factor in their relative vulnerability.

AD can present with multiple proteinopathies, including α-synuclein^18^ and TDP-43 inclusions^23^. While there are not yet well-established biomarkers for these pathologies, ^18^F-FDG PET studies have provided patterns probabilistically related to both entities^19–22^. We emphasize that this analysis was exploratory and requires further comprehensive validation. The cortical susceptible group harbored higher cingulate island ratio and worse visuospatial processing and trended toward greater frequency of hallucinations, all supportive of concomitant LBD^18,19^. In contrast, the limbic susceptible group had the greatest average age among groups (78.9 ± 6.1 years) and a pattern of MTL-predominant hypometabolism with asymmetry, all features that have been associated with LATE^21,22^. Parallel to semantic and episodic memory impairment associated with TDP-43 pathology^36–38^, the limbic susceptible group had the worst categorical and naming fluency. While these features are correlative and not comprehensive, the convergence of imaging, cognitive and clinical evidence support a potential contribution of copathology to susceptible groups with greater hypometabolism than expected given their level of T.

Resilience and susceptibility as defined here by N_M_ < T and N_M_ > T, respectively, may be thought of as a combination of separate yet related features, including relative levels of copathology and factors that directly influence the neuronal and glial responses to T pathology. Currently, it is more straightforward to assess the former, but the latter may reflect intrinsic resilience or vulnerability to T, perhaps related to genetic/epigenetic factors. Our copathology analyses suggest that susceptible groups have mixed cognitive impairment with more evidence of copathologies than the canonical group to contribute to hypometabolism not accounted for by T alone. Given the frequency of mixed disease on autopsy^16^, non-AD pathologies may represent an orthogonal axis along which canonical groups have intermediate levels of copathology, while resilient and susceptible groups have less or more mixed pathology, respectively (Fig. 5 and Supplementary Fig. 11). In the context of AD, these copathologies may be synergistic as non-AD proteinopathies can influence how neurons and glia respond to T^39–41^. Additional differences in non-disease related genetic, lifestyle and environmental factors also decouple the T/N_M_ relationship, representing attributes that affect how neurons respond to injury perhaps related to or distinct from copathology. The metabolic and cognitive profiles in resilient and susceptible groups were shared across A+ and A− cohorts and in symptomatic and asymptomatic patients. The observations of similar patterns of T/N_M_ mismatch and impaired cognition between A+ and A− susceptible groups are expected, as factors influencing the T/N_M_ relationship in AD may also be present in non-AD symptomatic patients. Given current constraints of in vivo biomarkers, autopsy data must confirm these hypotheses regarding specific copathology.

The study had several limitations. First, neuropathological validation is important for this work, but currently no datasets with tau PET, ^18^F-FDG PET and autopsy were available to us. Analyses in symptomatic individuals were performed on one cohort (ADNI) which includes multiple sites but with well-established data harmonization methods. Given the ADNI inclusion/exclusion criteria, this sample may not be representative of the broader population of cognitively impaired patients that harbor more mixed pathology, particularly vascular disease. A more heterogeneous sample might show more phenotypes/groups. However, the HABS dataset offers corresponding evidence of T/N_M_ dissociation patterns in cognitively normal older adults known to harbor significant regional relationships between tau and ^18^F-FDG PET^42^. Notably, these similar T/N_M_ groups arose with use of two distinct processing methods (ANTs for ADNI data and FreeSurfer for HABS data), indicating robustness of clustering to specific processing pipelines. Despite this, the separation of susceptible groups by imaging and cognitive factors associated with copathology highlight the non-trivial amount of copathology in ADNI participants. To this point, autopsy study of an ADNI subset demonstrated that α-synuclein and TDP-43 polypathology are frequently present in ADNI patients and correlate with antemortem imaging markers^16^. The canonical group is a statistical designation and does not quantify the absolute amount of AD vs. non-AD pathology. However, the canonical group does provide a relative benchmark for the population. While there was not much available data to study resilience-related factors, our initial analysis of resilient and susceptible groups supports the continued search for genetic, epigenetic and pathophysiological features that influence these relationships. Note that resilient groups had significantly higher APOE2 carrier frequency and the susceptible groups trended toward higher APOE4 frequency, though these differences were not seen after adjusting for A status (Supplementary Fig. 9B). Investigations into additional AD-associated features, such as glial and immune cell-mediated inflammation as well as blood brain barrier dysfunction, may also be warranted^4,43,44^.

Despite these limitations, the T/N_M_ mismatch approach may hold utility for biomedical research, specifically allowing clinical trials to measure heterogeneity across the Alzheimer’s continuum. For instance, the group of N_M_ > T susceptible patients may have mixed pathologies, which could reduce study power and complicate the assessment of investigational treatments designed to target single pathways. Consequently, future trials for anti-amyloid or anti-tau therapies might intentionally recruit patients or stratify findings based on T/N_M_ groups.

Overall, we define PET-based T/N_M_ mismatch measurements to evaluate the varying relationships of neuronal metabolism to T pathology in participants with cognitive decline. Dissociation in the T/N_M_ relationship demonstrates distinct groups with some showing resilience and others depicting susceptibility to T in terms of regional distributions of hypometabolism, cognition and pathological factors. T/N_M_ mismatch provides a quantitative spatial approach to assess neuroanatomical patterns of metabolic states affected by T pathology. This may improve our understanding of the biology and prognostication of subgroups in the Alzheimer’s and non-AD continuums. Additional studies may elucidate the heterogeneity of cellular metabolic responses to AD features as a step toward the successful implementation of precision medicine in AD.

## Methods

### Patient cohort

From the ADNI cohort database (http://adni.loni.usc.edu), we included participants with a ^18^F-flortaucipir (tau) PET and ^18^F-FDG PET performed within 1 year of each other, along with a measure of amyloid (A) status and a MRI scan (within about 1 year of PET scans). Of these, 289 participants with a diagnosis of mild cognitive impairment (MCI) or dementia were found. Evaluation of A status utilized ^18^F-florbetapir (*n* = 182) or ^18^F-florbetaben (*n* = 105) (amyloid) PET or Elecsys cerebrospinal fluid (CSF) Aβ assay (*n* = 2). Median time between ^18^F-FDG vs. tau PET in the ADNI cohort was 12 days (80% of cases within 1 month). Stratification by A enables analysis of T/N_M_ mismatch in patients along the Alzheimer’s continuum (A+, *n* = 164) and those with likely non-AD (A−, *n* = 125) pathology. Additional cohort details are listed in Supplementary Table 1. In the cognitively normal HABS cohort (data release 2.0; https://habs.mgh.harvard.edu/)^45^, we included 115 participants with tau PET, ^18^F-FDG PET, ^11^C-Pittsburgh compound B (amyloid) PET and MRI with the same criteria as above. The median time between ^18^F-FDG vs. tau PET in the HABS sample was 105 days (63% of cases within 5 months). See details in Supplementary Table 3. For the ADNI data, human subjects approval was obtained by the ADNI investigators to comply with the Institutional Review Board at each participating ADNI site. All participating ADNI sites received approval from their site’s Institutional Review Board; a complete listing of ADNI sites is provided at the end of the article file. All ADNI participants provided written informed consent. ADNI data was accessed according to the policies of the ADNI data sharing and publications committee. For the HABS data, HABS protocols were approved by the Partners Human Research Committee, the Institutional Review Board for the Massachusetts General Hospital and Brigham and Women’s Hospital, and all participants gave informed consent. HABS data was accessed according to the policies of the HABS data committee.

### Imaging data

Post-processed PET images from the ADNI data archive (http://adni.loni.usc.edu/data-samples/access-data/) were obtained^46^. Tau PET imaging was originally performed using the ADNI protocol with 30-min brain scans (six 5-min frames) starting 75 min after intravenous administration of ~10.0 mCi ^18^F-Flortaucipir. ^18^F-FDG PET imaging consisted of a 30-min scan (six 5-min frames) at 30 min after 5.0 mCi ^18^F-FDG injection. For amyloid PET, a 20-min brain scan (four 5-min frames) was performed 50 min after ~10.0 mCi ^18^F-Florbetapir or 90 min following ~8.1 mCi ^18^F-Florbetaben injection. Processed PET images with uniform isotropic resolution (8 mm full-width-at-half-maximum) were obtained with the ADNI archive description “Coreg, Avg, Std Img and Vox Size, Uniform Resolution.” ADNI MRI included a T1-weighted structural scan (resolution 1.0 × 1.0 × 1.2 mm^3^) and fluid attenuated inversion recovery (FLAIR) sequence MRI scan were acquired in the same session. For the HABS cohort^42,45^, we accessed spreadsheets of tau SUVR, ^18^F-FDG SUVR and ^11^C-Pittsburgh compound B distribution volume ratio.

### Image processing and PET regional analysis

MRI studies were processed using the ANTs (v2) pipeline^47^ for inhomogeneity correction, brain extraction, template registration and cortical thickness measurement^48,49^. MRI scans were divided into 104 cortical and subcortical ROIs with multi-atlas segmentation^50,51^ (http://neuromorphometrics.com/ParcellationProtocol_2010-04-05.PDF). PET images were co-registered to T1-weighted MRI with ANTs using rigid-body transformation^47^. SUVR maps were generated by convert3D (v1.1.0) with reference regions specific for each tracer: inferior cerebellar cortex for ^18^F-Flortaucipir^52^, cerebellar cortex for ^18^F-FDG^53^ and cerebellum for ^18^F-Florbetapir or ^18^F-Florbetaben^54,55^. Mean regional T and N_M_ measures were extracted from tau and ^18^F-FDG SUVR maps. Amyloid status (A+/A−) was determined with ^18^F-Florbetapir SUVR ≥ 1.11 or ^18^F-Florbetaben SUVR ≥ 1.08 computed from a composite ROI from middle frontal, anterior cingulate, posterior cingulate, inferior parietal, precuneus, supramarginal, middle temporal and superior temporal cortex^54,55^. Comparison between our amyloid SUVRs with available amyloid SUVRs from ADNI SUMMARYSUVR_WHOLECEREBNORM (UCBERKELEYAV45_01_14_21 and UCBERKELEYFBB_01_14_21, accessed 8/2021) revealed a strong correlation between amyloid measurements with *R*^2^ = 0.973 and slope *β* = 0.987 with no change in results. Two cases without amyloid PET had CSF amyloid-β_42_ < 980 pg/ml, meeting the threshold for A+ classification^56^. Braak staging was from separate processed ADNI data (UCBERKELEYAV1451_11_16_21, UCBERKELEYAV1451_PVC_11_16_21, accessed 11/2021) and thresholds (for Braak stages 1/2, 3/4, 5/6) derived from decision trees of tau SUVRs^57^. The HABS data consisted of amyloid status, tau and ^18^F-FDG SUVR spreadsheets in 84 cortical regions with FreeSurfer (v6) as generated in^42,45^ (data release 2.0; accessed 11/2021).

### Definition of regional T/N_M_ mismatch by clustering

Spatial patterns of T/N_M_ mismatch were investigated by clustering of the residuals on a regression model of ^18^F-FDG vs. tau SUVR. Robust linear regressions of individual ^18^F-FDG SUVR vs. a log transform of tau SUVR (to ameliorate effects of a skewed distribution of T) across all patients were performed in each of the 104 gray matter ROIs (Fig. 1) to yield T/N_M_ mismatch residuals (in units of ^18^F-FDG SUVR). A bi-square weighting function minimized the influence of outliers in robust regression. To attenuate the effect of outliers on clustering, regression residuals for each ROI and individual were discretized into a vector based on whether the residual was greater than 0.6 SD from the regression line (a cutpoint that identifies the farthest ~25% of points above or ~25% of points below the regression line) and if the residual was negative or positive, generating an array of 104 ROIs across 289 participants where each entry was −1, 0, or 1. Discretized residuals were inputs for Ward’s agglomerative hierarchical clustering^58^ with the hclust and cluster packages on R (v4.0.5) to create T/N_M_ mismatch groups. The number of clusters was selected by elbow and silhouette analysis^59^, which both suggested that *k* = 6 clusters optimizes within-cluster similarity. These methods did not agree on lower values, which would not capture as much between-group variation in specific regional patterns. Dimensionality reduction on discretized residuals was performed by PCA (Fig. 1) and *t*-SNE (Supplementary Fig. 1A). Regional mean residuals were visualized in cohort-based heatmaps, brain maps and three-dimensional renderings by ITK-SNAP^60^ and MRIcroGL^61^. Clustering validation was performed across 10-folds of 150 randomly selected ADNI participants, which showed stable group patterns and identities.

### Cognitive evaluation

ADNI and HABS performed cognitive testing using unified methodologies (accessed 8/2021 and 11/2021, respectively). We selected cognitive testing sessions closest to the ^18^F-FDG scan along with longitudinal follow-up testing. Global measures included AD Assessment Scale-Cognition 13 item (ADAS, higher score is worse)^62^, Clinical Dementia Rating sum of boxes (CDR-SOB, higher is worse)^63^ and Mini-Mental Status Exam (MMSE, lower is worse)^64^. Exploratory analysis was pursued with additional measures based on mismatch group findings and included the use of Clock Drawing Test^65^, NPI^66^ item B for proportion of patients with hallucinations after scan, ADNI *z*-scores for visuospatial, language and memory domains^67,68^, categorical fluency of animals^69^, Everyday Cognition test^70^ and Multilingual Naming Test^71^.

### Exploratory assessment of features associated with brain copathologies

Available vascular risk factors assessed at initial medical history were obtained from ADNI (INITHEALTH, accessed 4/2021), including presence of hypertension, hyperlipidemia, type 2 diabetes, arrhythmia, cerebrovascular disease, endovascular management of head/neck vessels, coronary artery disease (angina, stenosis, infarct), coronary interventions (stent, bypass graft), heart failure, structural heart defects and peripheral artery disease^44^. Number of subcortical infarcts (>3 mm in size) were centrally measured from MRI scans^72^ performed up to ^18^F-FDG scan (MRI_INFARCTS_01_29_21, accessed 4/2021). Infarcts mostly localized to cerebral white matter, basal ganglia and cerebellum. White matter hyperintensity (WMH) volumes were drawn from ADNI analysis of FLAIR MRI^73^ (ADNI_UCD_WMH_DICT_09_01_20, accessed 1/2021). Apolipoprotein E (*APOE*) allele frequency was analyzed (APOERES, accessed 7/2021).

In follow-up analyses, we calculated ^18^F-FDG PET measures which are thought to map to different non-AD pathologies. The cingulate island sign represents metabolic sparing of posterior cingulate cortex relative to precuneus and cuneus and has been associated with a-synuclein pathology. It was quantified as the ratio of posterior cingulate/precuneus/cuneus ^18^F-FDG SUVR; higher cingulate island ratio is linked to α-synucleinopathy^19,20^. The presence of TDP-43 pathology has been associated with MTL and FSO hypometabolism relative to inferior temporal gyrus (I). The I/MTL/FSO ratio was calculated as the ratio of inferior temporal gyrus/MTL/FSO gyrus ^18^F-FDG SUVR. Higher I/MTL/FSO ratio is associated with TDP-43-related disease^21,22^. An MTL asymmetry index was computed as |left−right|/(left+right) for ^18^F-FDG SUVR and cortical thickness^24^ as an additional potential marker of TDP-43 pathology^23^.

### Statistical analysis

Statistical analysis was performed in R (v4.0.5). All statistical tests were two-sided. Comparisons for variables such as cognition or tau and ^18^F-FDG SUVRs were performed with likelihood ratio tests by linear regression. Covariates included sex, age, education, amyloid status (A+/A−) and tau SUVR in the inferior temporal gyrus, a region where T correlates with disease severity^5,17^. Multiple test adjustment by Benjamini–Hochberg correction with false discovery rate = 0.05 was conducted for pairwise comparisons with the canonical group. Box plots show the data points as dots, mean as an X symbol, median as the middle box line, first quartile (Q1) and third quartiles (Q3) as box edges (denoting the interquartile range, IQR), whiskers as the minimum/maximum points and outliers based on thresholds <Q1 − 1.5(IQR) or >Q3 + 1.5(IQR). Exploratory analyses (such as for copathology biomarkers) were also performed without multiple test adjustment. Genotype frequency comparisons were performed with *χ*^2^ tests. Longitudinal cognitive trajectories were assessed with linear mixed effects models to account for participant-specific random intercepts with baseline cognitive score at scan, time from scan, cluster and cluster*time interaction as independent variables and sex, age, education and A status as covariates. Slopes of annual cognitive change for each cluster were defined as the sum of the time from scan slope and cluster*time interaction slope. Differences in decline rates were assessed by significance of the slope of the cluster*time interaction.

### Reporting summary

Further information on research design is available in the Nature Research Reporting Summary linked to this article.

## Supplementary information

Supplementary Information

Reporting Summary

Description of Additional Supplementary Files

Supplementary Data 1

## Data Availability

The raw and processed data including the participant scans and spreadsheets described above are available on the data archives of the Alzheimer’s Disease Neuroimaging Initiative (ADNI) (http://adni.loni.usc.edu) and the Harvard Aging Brain Study (https://habs.mgh.harvard.edu/)^45^. Supplementary Material is available online. Additional information can be provided by the authors upon reasonable request. Source data are provided with this paper.

## Source data

Source Data

## Code availability

Relevant code can be found at: http://stnava.github.io/ANTs/. This includes links to scripts on brain extraction (https://github.com/ANTsX/ANTs/blob/master/Scripts/antsBrainExtraction.sh), image registration (https://github.com/ANTsX/ANTs/tree/master/ImageRegistration) and segmentation (https://github.com/ANTsX/ANTs/tree/master/ImageSegmentation). Additional information can be provided by the authors upon reasonable request.
