# Supplementary material for: Dissociation of tau pathology and neuronal hypometabolism within the ATN framework of Alzheimer’s disease
Source: Nat Commun. 2022 Mar 21;13:1495. doi: 10.1038/s41467-022-28941-1 (PMC8938426; doi:10.1038/s41467-022-28941-1)
Supplement: Supplementary file 1 — Supplementary Information [file 41467_2022_28941_MOESM1_ESM.pdf]

SUPPLEMENTARY INFORMATION

Supplementary Table 1 Cohort characteristics of the 289 cognitively impaired patients from the AD Neuroimaging Initiative (ADNI) study. Frequencies are listed for diagnosis (Mild Cognitive Impairment (MCI)/dementia) and sex (F/M). Mean (standard deviation) values are shown for age and education (in years), AD Assessment Scale Cognition (ADAS-Cog, higher is worse), Clinical Dementia Rating Sum of Boxes (CDR, higher is worse), Mini-Mental Status Examination (MMSE, lower score is worse) and mean global cortical thickness (mm).

A± Status	MCI/ Dementia	F/ M	Age	Educ	Cognition			Cortical Thickness (mm)
					ADAS-Cog	CDR-SOB	MMSE	
A+	102	75	74.9	15.9	25.0	2.8	25.7	1.87
(n=164)	62	89	(9.5)	(2.6)	(8.5)	(2.2)	(3.5)	(0.44)
A–	112	49	73.8	16.2	18.1	1.8	28.0	2.02
(n=125)	13	76	(8.5)	(2.8)	(6.3)	(1.7)	(2.0)	(0.41)

Supplementary Table 2 T/N$_M$ mismatch clustering across A– patients. Diagnosis (MCI/dementia) and sex (F/M) are in frequencies. Mean (standard deviation) values are shown for age/education (years), AD Assessment Scale-Cognitive (ADAS-Cog, higher is worse), Clinical Dementia Rating scale sum of boxes (CDR-SOB, higher is worse), Mini-Mental Status Exam (MMSE, lower is worse) and global cortical thickness (mm). The last row depicts group difference *P* values by likelihood ratio tests after adjusting for covariates. Covariates include sex, age, education and inferior temporal gyrus tau SUVR.

Group	MCI/ Dem	F/ M	Age (y)	Educ (y)	Cognition			Cortical Thickness (mm)
					ADAS-Cog	CDR-SOB	MMSE	
High Cortical Resilient	26/1	13/14	73.7 (8.6)	16.0 (2.7)	17.1 (4.8)	1.7 (1.3)	28.3 (2.5)	2.12 (0.43)
Limbic Resilient	9/0	3/6	79.3 (7.5)	16.4 (2.9)	17.3 (5.3)	1.6 (1.1)	29.0 (1.1)	2.03 (0.26)
Low Cortical Resilient	26/0	12/14	69.1 (8.0)	16.5 (2.6)	16.2 (4.9)	1.2 (0.8)	28.5 (1.7)	2.12 (0.52)
Canonical	34/6	14/26	74.1 (8.2)	16.4 (2.6)	18.6 (7.4)	1.9 (2.2)	27.9 (2.5)	1.99 (0.39)
Cortical Susceptible	12/4	4/12	75.8 (7.7)	14.9 (3.7)	21.4 (6.5)	2.4 (2.1)	26.9 (2.1)	1.93 (0.25)
Limbic Susceptible	5/2	3/4	79.3 (8.4)	17.3 (2.8)	19.7 (7.1)	2.6 (2.2)	26.9 (2.3)	1.65 (0.31)
Group *P* val		0.40	0.50	0.49	0.18	0.54	0.16	0.61

Supplementary Table 3 Cohort characteristics of the 115 cognitively normal patients from the Harvard Aging Brain Study (HABS). Frequencies are listed for sex (F/M). Mean (SD) values are shown for age and education (in years), Clinical Dementia Rating Sum of Boxes (CDR, higher is worse), Mini-Mental Status Examination (MMSE, lower score is worse) and mean global cortical thickness (mm).

A± Status	F/ M	Age (y)	Educ (y)	Cognition		Cortical Thickness (mm)
				CDR-SOB	MMSE	
A+	20	78.5	15.8	0.23	28.9	2.42
(n=37)	17	(6.0)	(2.9)	(0.34)	(1.2)	(0.09)
A–	44	75.8	16.3	0.18	29.2	2.44
(n=78)	34	(7.0)	(3.0)	(0.30)	(1.2)	(0.09)

Supplementary Table 4 T/N_M mismatch clustering across HABS participants. Amyloid status (A–/A+) and sex (F/M) are in frequencies. Mean (standard deviation) values are shown for age/education (years), Clinical Dementia Rating scale sum of boxes (CDR, higher is worse), Mini-Mental Status Exam (MMSE, lower is worse) and global cortical thickness (mm). The last row depicts group difference *P* values by likelihood ratio tests after adjusting for covariates. Significant differences in pairwise comparisons between a non-canonical and canonical group with covariate adjustment are annotated. For pairwise comparisons, * denotes *P*<0.05 after multiple tests (Benjamini-Hochberg) adjustment and $^{+}$ denotes *P*<0.05 before multiple tests adjustment. Covariates include sex, age, education, A status and inferior temporal gyrus tau SUVR.

**Group**	**A+/ A–**	**F/ M**	**Age** (y)	**Educ** (y)	**Cognition**		**Cortical Thickness** (mm)
					**CDR-SOB**	**MMSE**	
High Cortical Resilient	4/9	9/4	77.3 (7.0)	16.1 (2.6)	0.12 (0.30)	28.9 (1.5)	2.44 (0.09)
Limbic Resilient	2/3	3/2	80.1 (8.8)	15.6 (4.1)	0.40 (0.55)	28.6 (1.7)	2.44 (0.08)
Low Cortical Resilient	3/16	12/7	75.8 (4.9)	16.6 (3.0)	0.16 (0.24)	29.4 (1.0)	2.45 (0.07)
Canonical	10/29	20/19	74.0 (6.7)	15.6 (2.8)	0.22 (0.38)	29.5 (0.9)	2.46 (0.08)
Cortical Susceptible	14/12	14/12	79.5 (6.4)*	16.5 (3.4)	0.19 (0.25)	28.7 (1.3)$^{+}$	2.39 (0.10)
Limbic Susceptible	4/9	6/7	78.0 (6.9)	16.3 (2.9)	0.19 (0.25)	28.7 (1.5)$^{+}$	2.41 (0.09)
Group *P* val		0.78	0.002	0.27	0.28	0.008	0.07

Supplementary Table 5 Summary of linear mixed effects models of baseline scores nearest to the ^{18}F-FDG scan and cognitive trajectories, with covariates, across all study participants. Baseline scores and slopes are shown for ADAS-Cog (higher is worse), CDR-SOB (higher is worse) and MMSE (lower is worse). Significant differences in pairwise slope comparisons with the canonical group were performed with linear mixed effects analysis with covariates after multiple test (Benjamini-Hochberg) adjustment are denoted **P*<0.05, ***P*<0.005. $^{+}$*P*<0.05 before multiple test adjustment. Covariates include sex, age, education, A status and T level. Sample sizes and *P* values are listed in Supplementary Data 1.

**Group**	**ADAS-Cog**		**CDR-SOB**		**MMSE**	
	Baseline	Slope	Baseline	Slope	Baseline	Slope
High Cortical Resilient	19.6	-0.07	1.8	0.05^{+}	27.8	-0.04
Limbic Resilient	20.0	0.57	2.0	0.53	27.1	-0.57
Low Cortical Resilient	18.8	0.61	1.7	0.03	27.7	-0.47
Canonical	22.9	0.77	2.5	0.46	26.7	-0.36
Cortical Susceptible	26.0	2.36**	3.5	0.68	25.1	-0.55
Limbic Susceptible	25.6	3.85**	3.2	1.70**	25.3	-3.08**

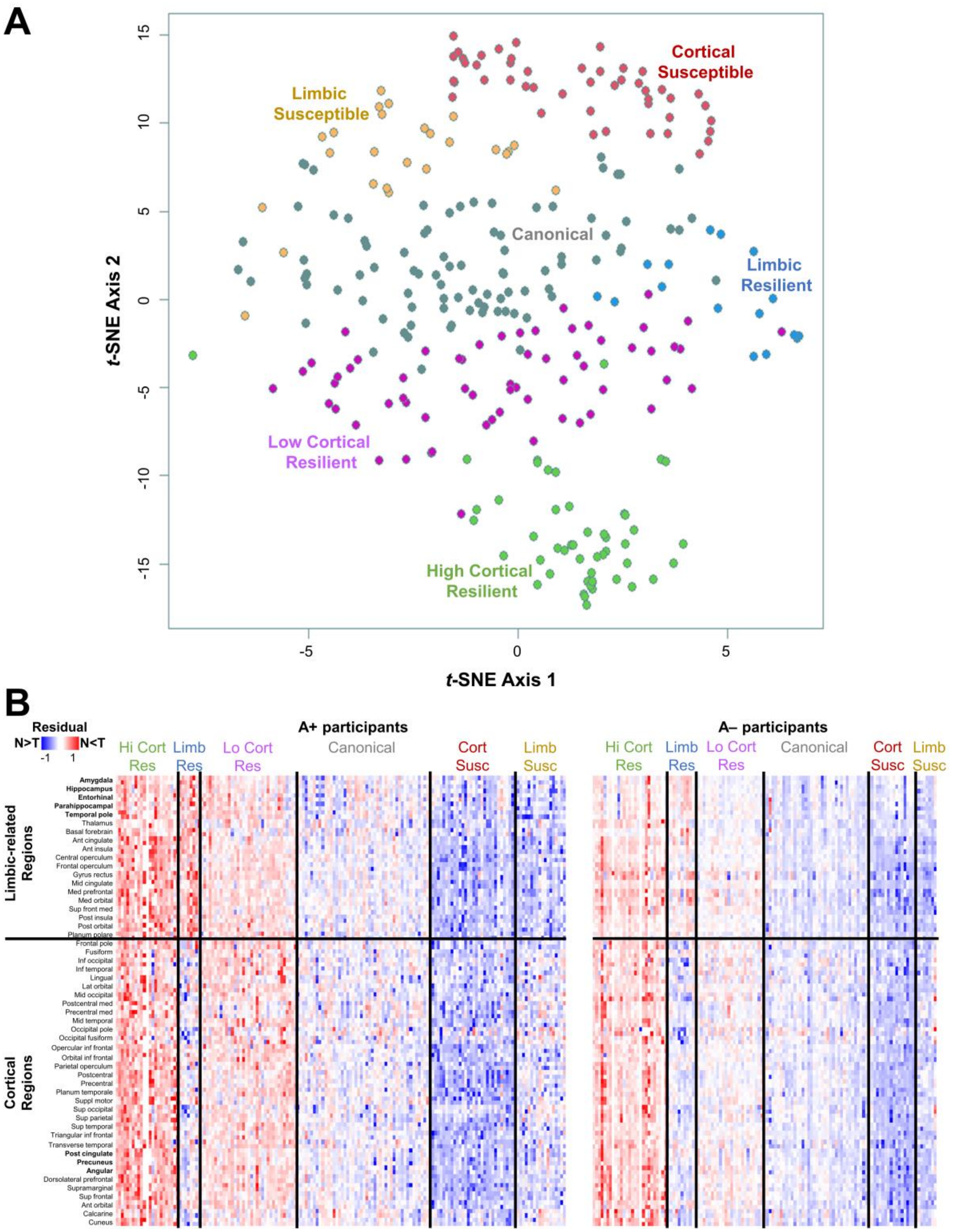

Supplementary Fig. 1 Analysis of T/N_M residuals and clusters. (A) Mapping of group identity onto a *t*-distributed stochastic neighbor embedding (*t*-SNE) plot. Axis 2 may correspond to the degree of T/N_M relation, with values near 0 as T~N, negative values as N_M<T resilient and positive values as N_M>T susceptible. Axis 1 may correspond to regional localization of N_M, with values near 0 as both limbic and cortical N_M, negative values greater limbic N_M with less cortical N_M and positive values as greater cortical N_M with less limbic N_M. (**B**) Heatmap of regional residuals of the T/N_M relationship for individual A+ (left) and A– (right) participants across groups and brain regions. Red denotes positive residuals (N_M<T). Blue denotes negative residuals (N_M>T).

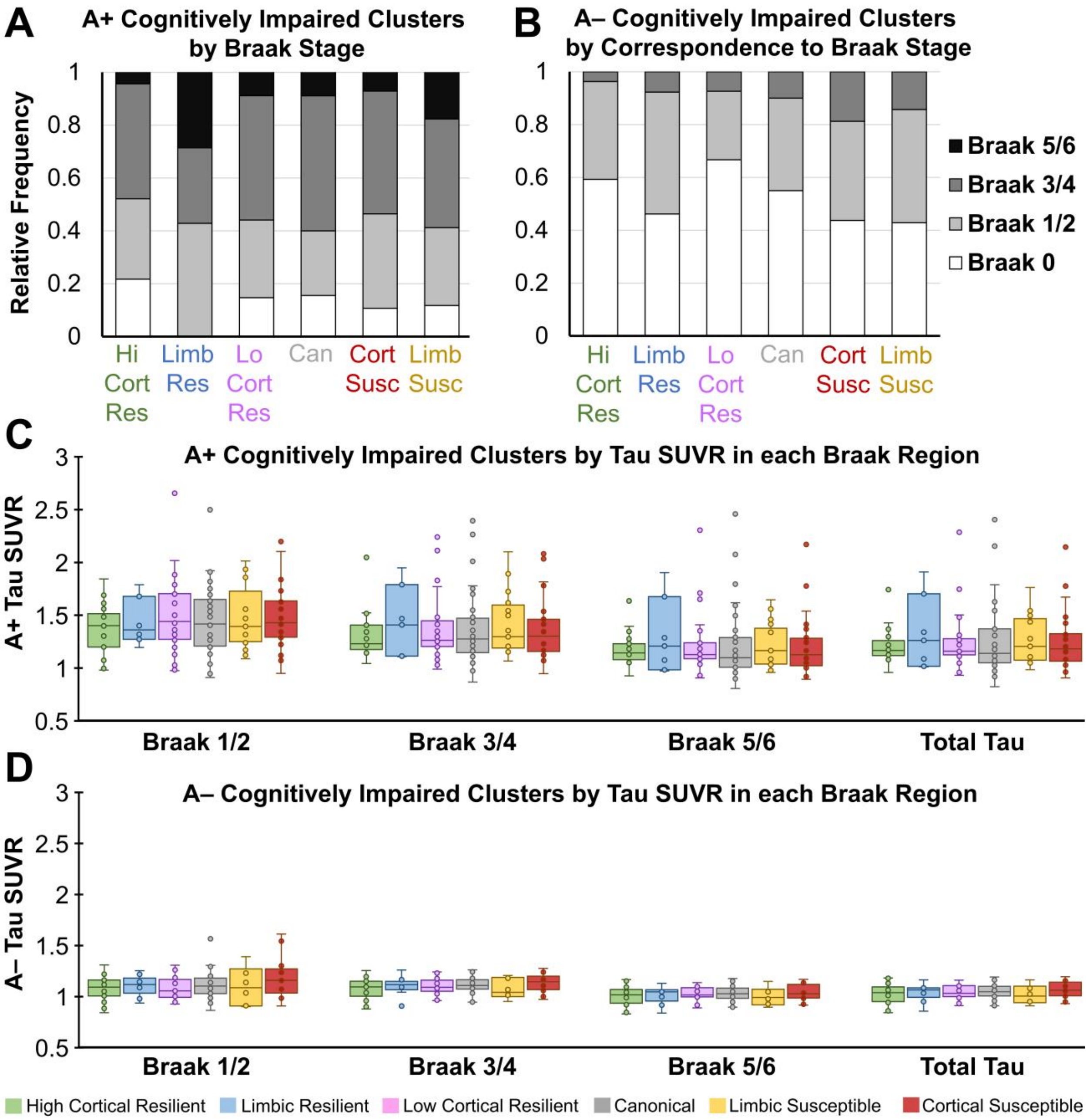

Supplementary Fig. 2 Distributions of tau pathology across groups. The distribution of tau PET uptake corresponding to AD Braak staging classification in (**A**) A+ and (**B**) A– individuals is shown (note that for A– individuals, these designations represent regional measures of tau pathology that would correspond to Braak stages in AD). Braak stage categories were compared by χ^2-goodness-of-fit tests, which showed no significant group differences. Tau PET SUVRs representing different Braak regions are similar across groups in (**C**) A+ and (**D**) A– individuals. Group differences in regional tau SUVRs was not significant. Sample sizes are provided in Supplementary Data 1. Source data are provided as a Source Data file.

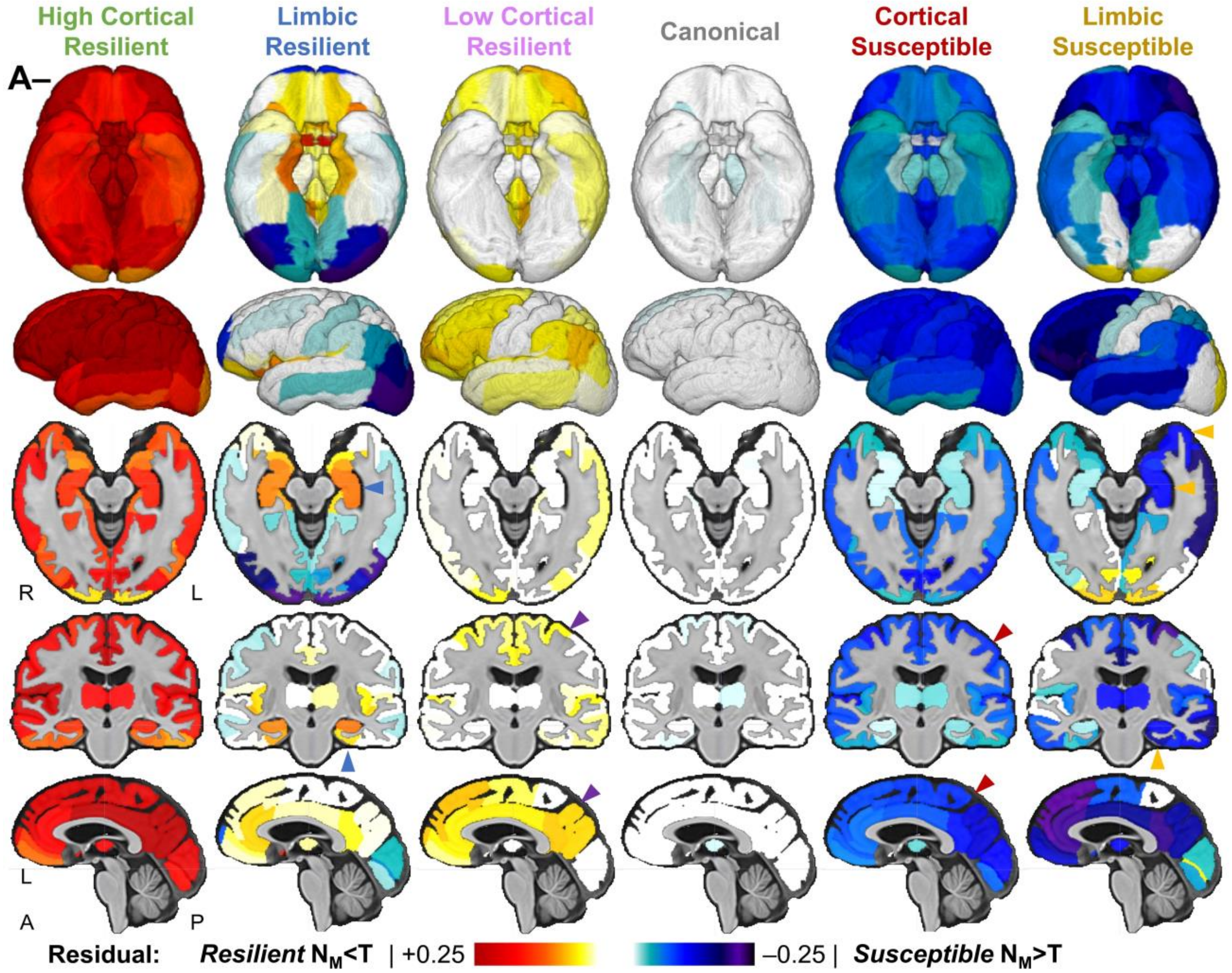

Supplementary Fig. 3 Three- and two-dimensional brain maps of T/N_M mismatch visualize T/N_M relationships and spatial patterns in A– participants. Mean T/N_M relation residuals for each region shown. Compared to the canonical (N_M~T) group, resilient (N_M<T) and susceptible (N_M>T) groups have limbic vs. cortical involvement (arrowheads). Abbreviations: right (R), left (L), anterior (A), posterior (P).

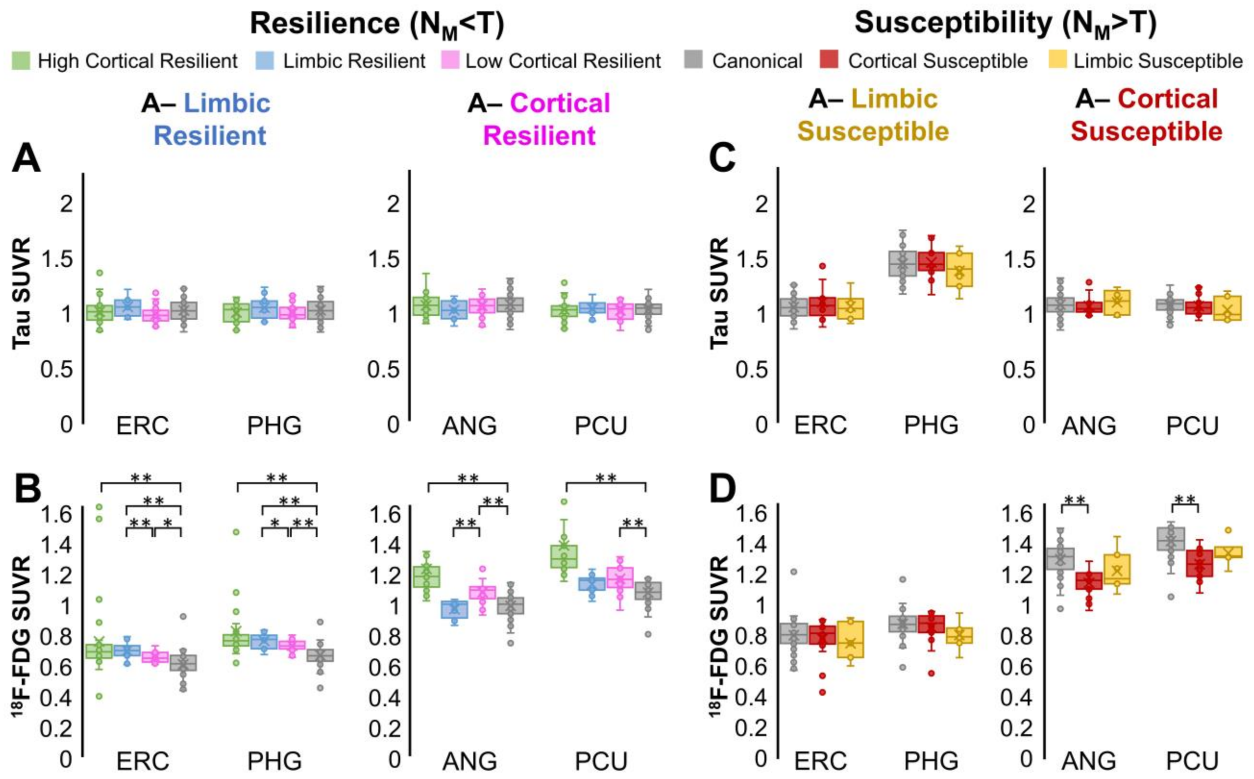

Supplementary Fig. 4 T/N_M mismatch depicts differences in regional ^{18}F-FDG but not tau SUVR in A– patients. Resilient groups had (**A**) similar regional tau SUVRs in limbic and cortical regions though (**B**) unique ^{18}F-FDG SUVRs in limbic and cortical regions. Susceptible groups had (**C**) similar regional tau SUVRs in limbic and cortical regions and (**D**) unique ^{18}F-FDG SUVRs in limbic and cortical regions. Example limbic regions: entorhinal cortex (ERC), parahippocampal gyrus (PHG). Example cortical regions: angular gyrus (ANG), precuneus (PCU). Box plots show data points as dots, mean as an "X" symbol, median as the middle box line, first quartile (Q1) and third quartiles (Q3) as box edges (denoting the interquartile range, IQR), whiskers as the minimum/maximum points and outliers based on thresholds <Q1–1.5(IQR) or >Q3+1.5(IQR). Significant differences in pairwise comparisons with the canonical group by two-tailed likelihood ratio tests after covariate and multiple test (Benjamini-Hochberg) adjustment are denoted as *$P<0.05$, **$P<0.005$. Covariates include sex, age, education and T level. Sample sizes and P values are provided in Supplementary Data 1. Source data are provided as a Source Data file.

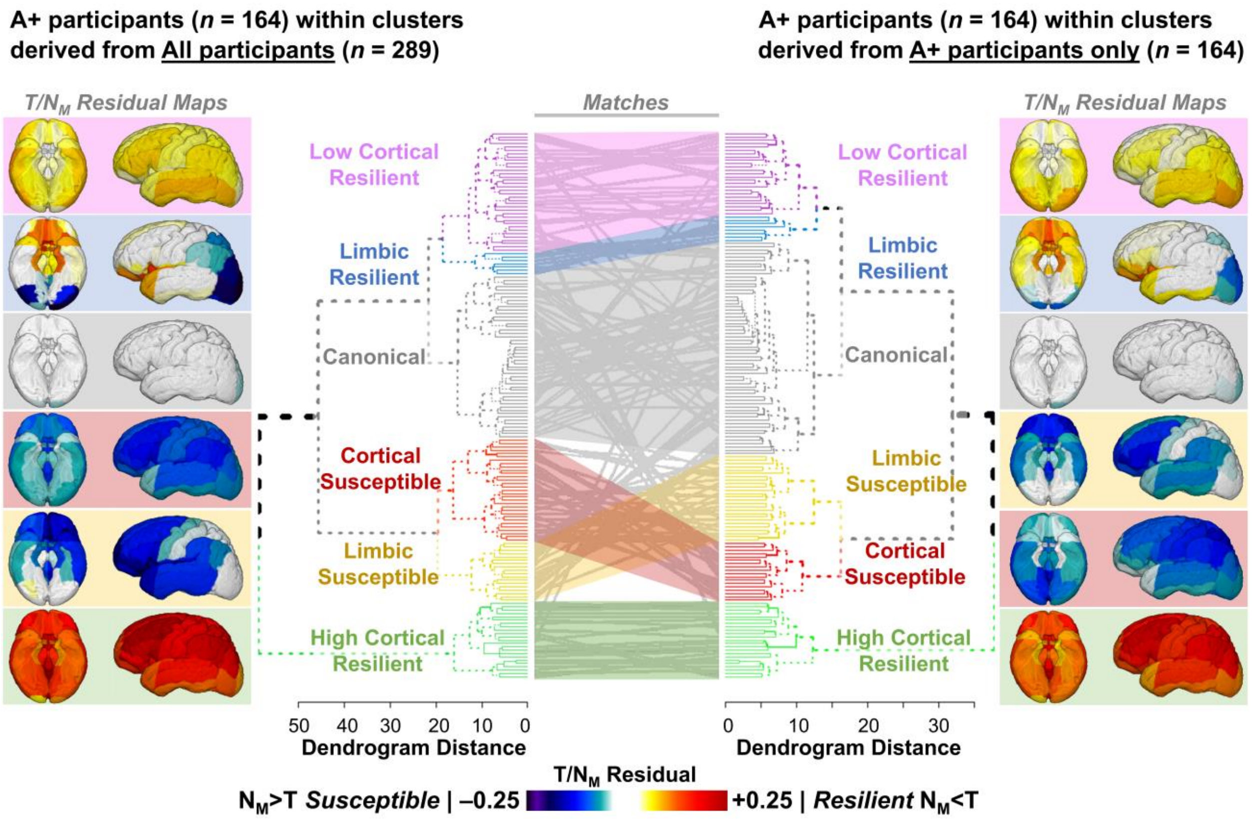

Supplementary Fig. 5 Group identities of A+ participants are similar when based on clustering of all participants or just A+ participants. Group identity and patterns of A+ participants (*n* = 164) when clustering on all participants (*n* = 289) (left-hand side) are similar to group identity and patterns of A+ participants when clustered alone (right-hand side). Dendrograms, individual matches and three-dimensional renderings of T/N_M residual maps are shown. Note that A– participants were included in the clustering process on the left-hand side (all participants) but are not visualized on the left-hand side dendrogram because they were intentionally excluded from clustering on the right-hand side dendrogram.

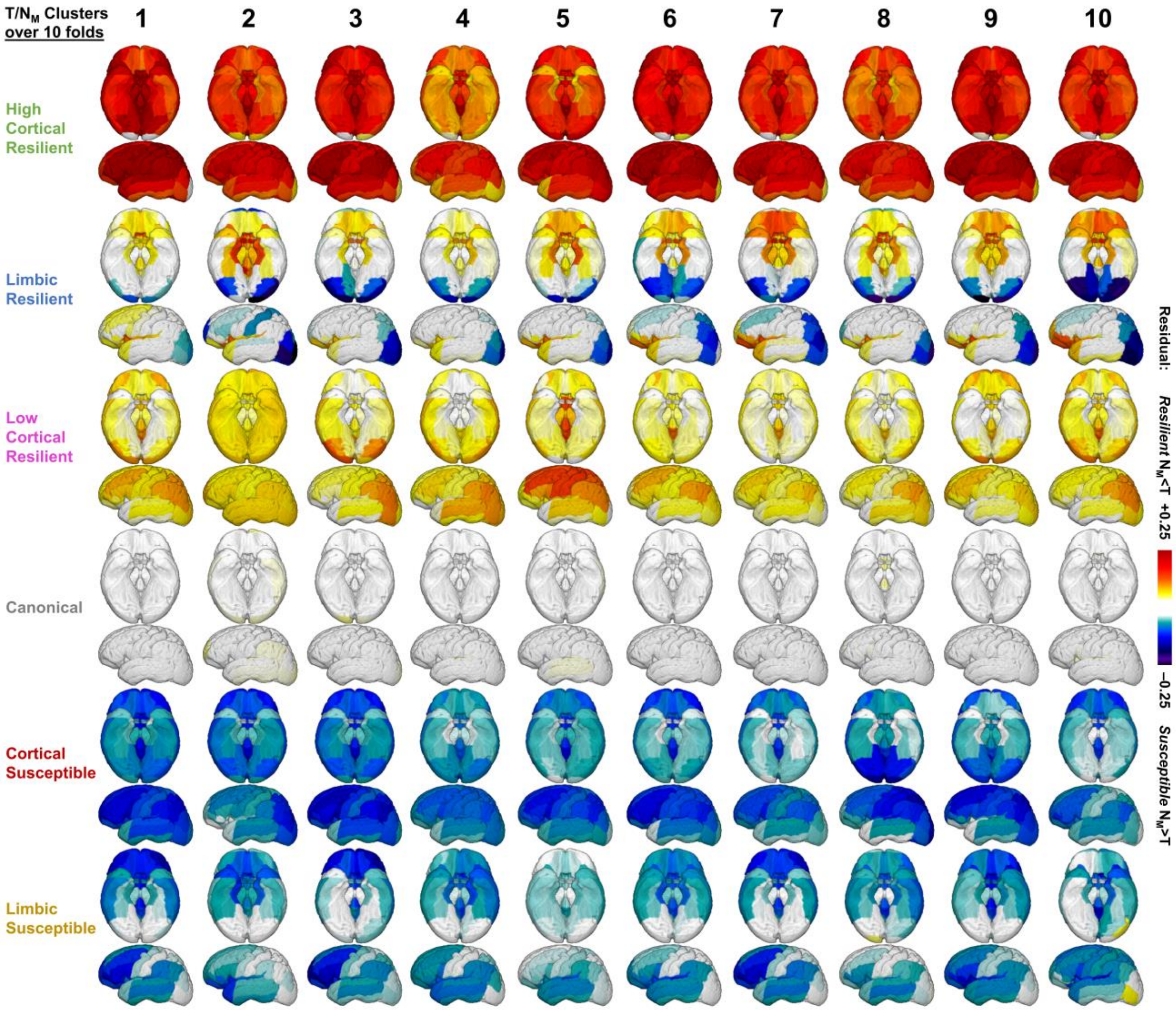

Supplementary Fig. 6 Clustering is stable across 10 folds of 150 randomly selected participants. Rows show the distinct group patterns across 10 folds, where each column represents one fold. About 90% of participants had their original group identity match with the majority-fold group identity while about 9% of participants had changes in group identity calls in the same residual direction (such as a change from one cortical resilient group to another).

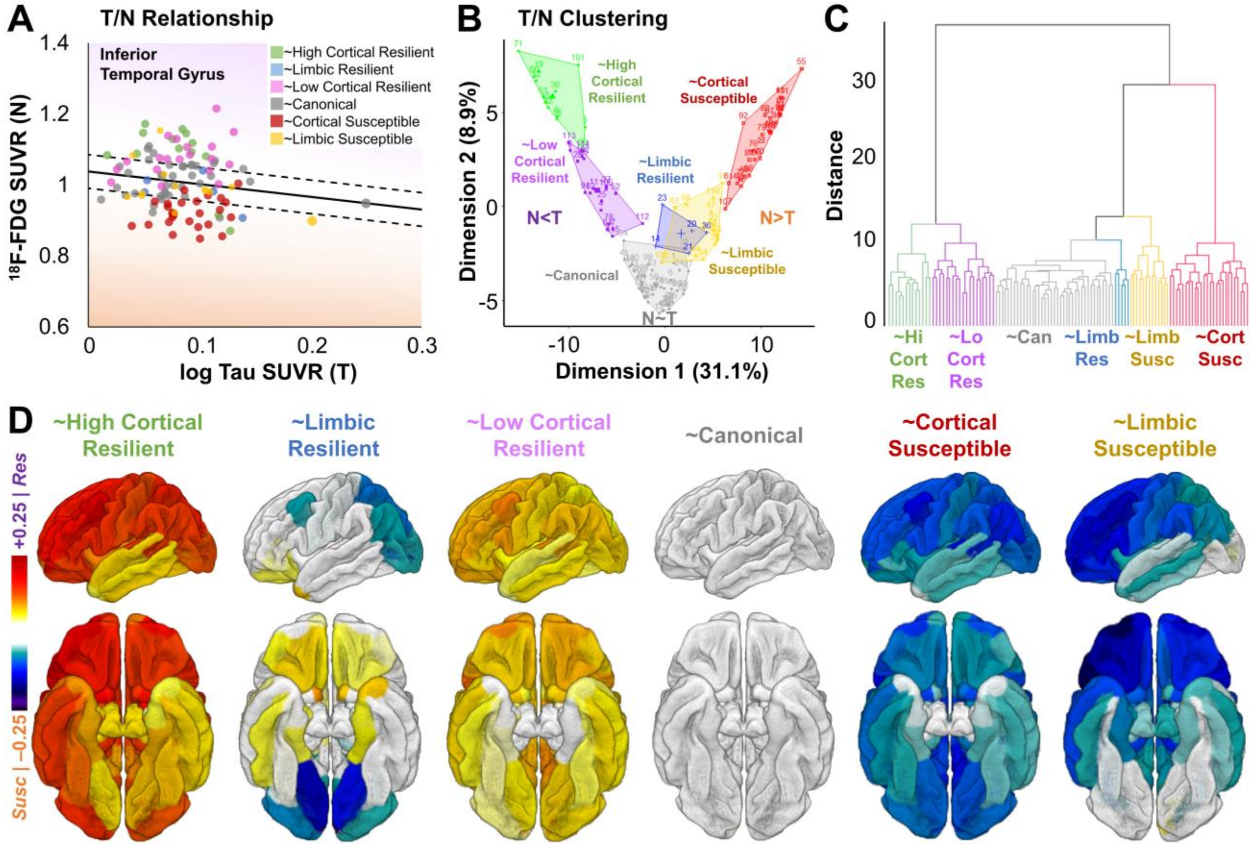

Supplementary Fig. 7 T/N$_M$ clustering on 115 cognitively normal participants in the Harvard Aging Brain Study (HABS) reveals T/N$_M$ patterns similar to those seen in symptomatic ADNI participants. (**A**) A regression model of ^{18}F-FDG vs. tau SUVR in the inferior temporal gyrus, a typical tau staging region in AD is displayed as a representative region. Solid line represents the model fit, with dashed lines denoting standard deviation-based thresholds. Above the model line (purple background) are participants with N$_M$<T in this ROI while below the line (orange background) are participants with N$_M$>T. Source data are provided as a Source Data file. Clustering by T/N$_M$ residuals of all regional and patient residuals is visually demonstrated by (**B**) principal component analysis and (**C**) dendrogram. (**D**) Three-dimensional maps visualize T/N$_M$ mismatch relationships and spatial patterns of mean T/N$_M$ relation regional residuals, which are visually similar to results from **Fig. 2**.

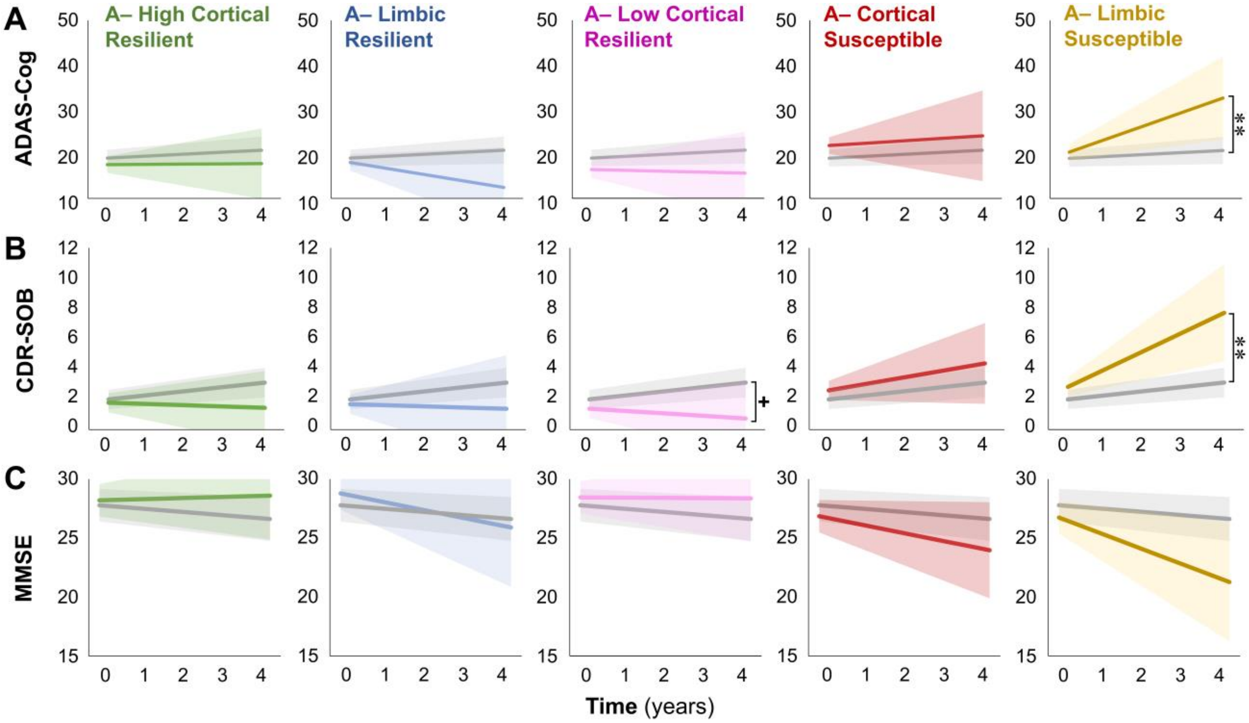

Supplementary Fig. 8 T/N_M mismatch portrays differential cognitive decline in A– participants. Tests include (**A**) AD Assessment Scale Cognitive 13 item (ADAS-Cog), (**B**) Clinical Dementia Rating Sum of Boxes (CDR-SOB) and (**C**) Mini-Mental Status Examination (MMSE) by linear mixed effects models with amyloid status, baseline score, education, sex and age as covariates. Lines show the mixed effect model and error bands show ± 1 propagated standard error. Significant differences in pairwise comparisons of cognitive decline between a non-canonical and canonical group by linear mixed effects analysis with multiple test (Benjamini-Hochberg) adjustment are denoted as $^{**}P<0.005$. $^{+}$ denotes $P<0.05$ before multiple tests adjustment. Sample sizes and P values are provided in Supplementary Data 1. Source data are provided as a Source Data file.

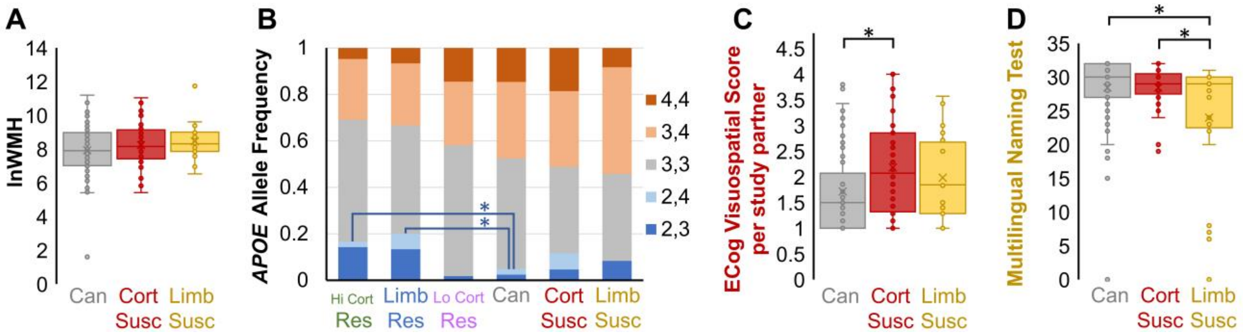

Supplementary Fig. 9 Additional exploratory features of T/N_M mismatch groups in the symptomatic ADNI cohort. (**A**) Both susceptible groups showed trends to higher log-normalized white matter hyperintensity volume (lnWMH in $\ln(\text{mm}^3)$)). (**B**) Distribution of Apolipoprotein E (*APOE*) allele frequency (ε2, ε3, ε4) across T/N_M mismatch groups in all participants. Significant differences were noted by χ^2 tests with * $P < 0.05$. Differences did not remain significant after adjusting for amyloid status among groups. (**C**) The cortical susceptible group had significantly worse visuospatial scores on the Everyday Cognition (ECog) per study partner evaluation. (**D**) The limbic susceptible group had significantly worse scores on the Multilingual Naming Test. Box plots show data points as dots, mean as an "X" symbol, median as the middle box line, first quartile (Q1) and third quartiles (Q3) as box edges (denoting the interquartile range, IQR), whiskers as the minimum/maximum points and outliers based on thresholds <Q1–1.5(IQR) or >Q3+1.5(IQR). Significant differences within cognitive testing was found by two-tailed likelihood ratio tests with amyloid status, education, sex and age as covariates. Sample sizes and P values are provided in Supplementary Data 1. Source data are provided as a Source Data file.

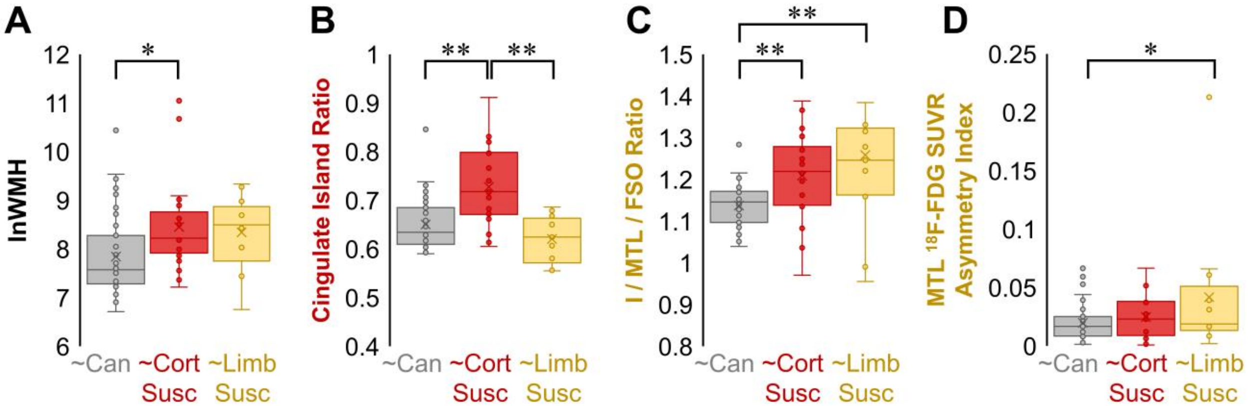

Supplementary Fig. 10 Exploratory analysis of non-AD pathologies in cognitively normal T/N$_M$ groups in the HABS cohort. (**A**) The group analogous to the cortical susceptible group had significantly higher log-normalized white matter T1-weighted hypointensity volume (lnWMH in ln(mm^3), analogous to FLAIR sequence white matter hyperintensity). The group analogous to the cortical susceptible group had higher (**B**) cingulate island ratio across groups. Susceptible groups had greater (**C**) inferior temporal/MTL/frontal supraorbital ^{18}F-FDG ratio (I/MTL/FSO ratio) and more (**D**) MTL asymmetry on ^{18}F-FDG SUVR. Box plots show data points as dots, mean as an "X" symbol, median as the middle box line, first quartile (Q1) and third quartiles (Q3) as box edges (denoting the interquartile range, IQR), whiskers as the minimum/maximum points and outliers based on thresholds <Q1–1.5(IQR) or >Q3+1.5(IQR). Significance was assessed by two-tailed likelihood ratio tests with amyloid status, education, sex and age as covariates as *P<0.05 and **P<0.005. These results were not corrected for multiple comparisons due to their exploratory nature. Sample sizes and P values are provided in Supplementary Data 1. Source data are provided as a Source Data file.

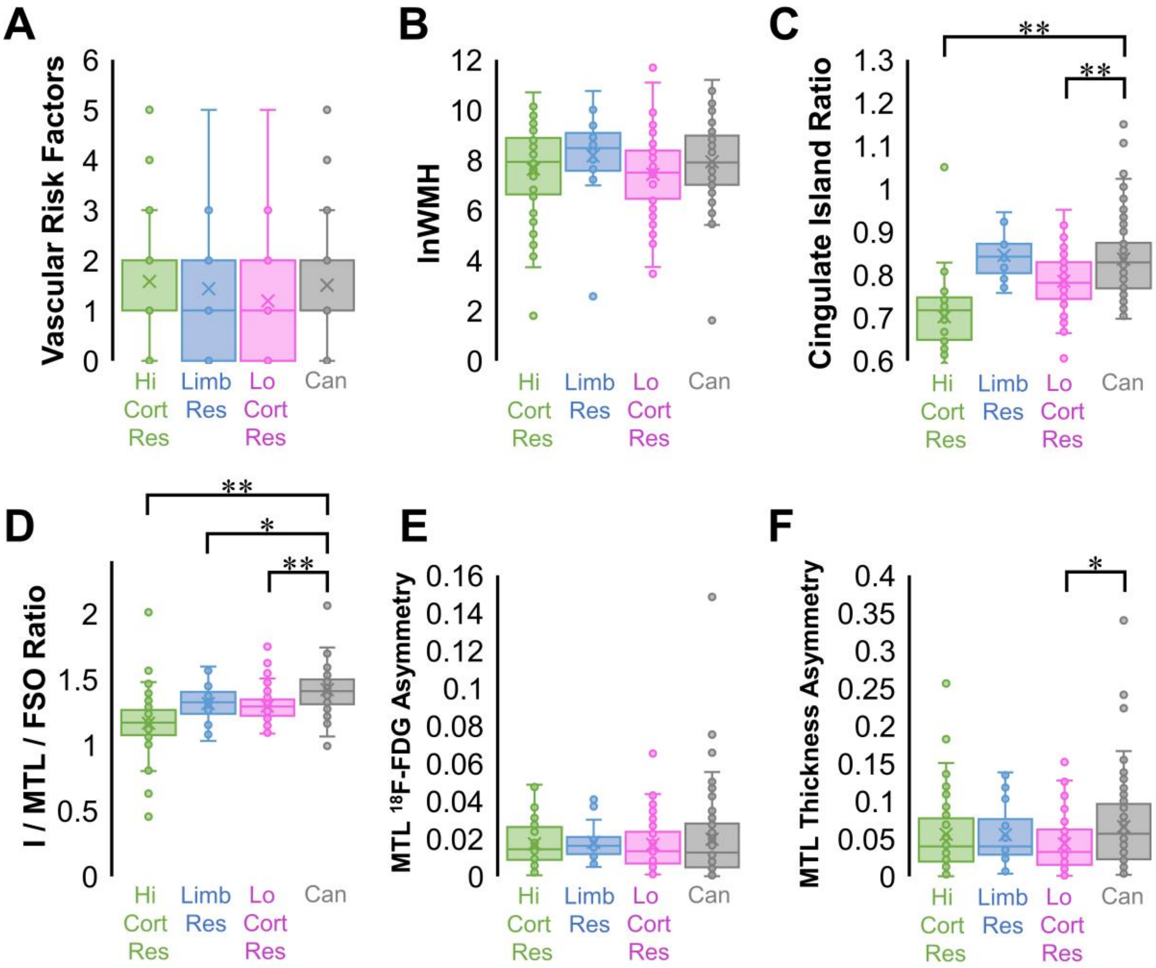

Supplementary Fig. 11 Exploratory analysis of vascular, α-synuclein (Lewy body) and TDP-43 copathology features in T/N$_M$ resilient groups from ADNI data. Canonical and resilient groups did not differ in (**A**) vascular risk factors and (**B**) log-normalized white matter hyperintensity volume (lnWMH in ln(mm^3)). High and low cortical resilient group had lower (**C**) cingulate island ratios. Resilient groups had less (**D**) inferior temporal/MTL/frontal supraorbital ^{18}F-FDG ratio (I/MTL/FSO ratio) and lower MTL asymmetry on (**E**) ^{18}F-FDG SUVR and (**F**) thickness. Box plots show data points as dots, mean as an "X" symbol, median as the middle box line, first quartile (Q1) and third quartiles (Q3) as box edges (denoting the interquartile range, IQR), whiskers as the minimum/maximum points and outliers based on thresholds <Q1–1.5(IQR) or >Q3+1.5(IQR). Significance was assessed by two-tailed likelihood ratio tests with amyloid status, education, sex and age as covariates as *P<0.05 and **P<0.005. These results were not corrected for multiple comparisons due to their exploratory nature. Sample sizes and P values are provided in Supplementary Data 1. Source data are provided as a Source Data file.
