# Supplementary material for: Dissociation of tau pathology and neuronal hypometabolism within the ATN framework of Alzheimer’s disease
Source: Nat Commun. 2022 Mar 21;13:1495. doi: 10.1038/s41467-022-28941-1 (PMC8938426; doi:10.1038/s41467-022-28941-1)
Supplement: Supplementary file 2 — Reporting Summary [file 41467_2022_28941_MOESM2_ESM.pdf]

nature portfolio

Corresponding author(s): David Wolk and Ilya Nasrallah

Last updated by author(s): Jan 24, 2022

Reporting Summary

Nature Portfolio wishes to improve the reproducibility of the work that we publish. This form provides structure for consistency and transparency in reporting. For further information on Nature Portfolio policies, see our Editorial Policies and the Editorial Policy Checklist.

Statistics

For all statistical analyses, confirm that the following items are present in the figure legend, table legend, main text, or Methods section.

n/a	Confirmed	
☐	☒	The exact sample size (*n*) for each experimental group/condition, given as a discrete number and unit of measurement
☐	☒	A statement on whether measurements were taken from distinct samples or whether the same sample was measured repeatedly
☐	☒	The statistical test(s) used AND whether they are one- or two-sided<br>*Only common tests should be described solely by name; describe more complex techniques in the Methods section.*
☐	☒	A description of all covariates tested
☐	☒	A description of any assumptions or corrections, such as tests of normality and adjustment for multiple comparisons
☐	☒	A full description of the statistical parameters including central tendency (e.g. means) or other basic estimates (e.g. regression coefficient) AND variation (e.g. standard deviation) or associated estimates of uncertainty (e.g. confidence intervals)
☐	☒	For null hypothesis testing, the test statistic (e.g. *F*, *t*, *r*) with confidence intervals, effect sizes, degrees of freedom and *P* value noted<br>*Give P values as exact values whenever suitable.*
☒	☐	For Bayesian analysis, information on the choice of priors and Markov chain Monte Carlo settings
☒	☐	For hierarchical and complex designs, identification of the appropriate level for tests and full reporting of outcomes
☒	☐	Estimates of effect sizes (e.g. Cohen's *d*, Pearson's *r*), indicating how they were calculated

Our web collection on statistics for biologists contains articles on many of the points above.

Software and code

Policy information about availability of computer code

Data collection	No software was used for data collection. Refer to ADNI and HABS data websites for details.
Data analysis	Neuroimaging processing of ADNI data with ANTs (v2), convert3d (v1.1.0). Preprocessed HABS data utilized Freesurfer (v6).<br>Clustering and statistical analysis with R (v4.0.5).<br>Imaging data visualization with ITK-SNAP (v3.8) and MRIcroGL (v1.2).

For manuscripts utilizing custom algorithms or software that are central to the research but not yet described in published literature, software must be made available to editors and reviewers. We strongly encourage code deposition in a community repository (e.g. GitHub). See the Nature Portfolio guidelines for submitting code & software for further information.

Data

Policy information about availability of data

All manuscripts must include a data availability statement. This statement should provide the following information, where applicable:

- Accession codes, unique identifiers, or web links for publicly available datasets
- A description of any restrictions on data availability
- For clinical datasets or third party data, please ensure that the statement adheres to our policy

Raw and processed data including the scans and spreadsheets described above are available on the data archives of the Alzheimer's Disease Neuroimaging Initiative (ADNI) (http://adni.loni.usc.edu) and the Harvard Aging Brain Study (https://habs.mgh.harvard.edu/). The data request forms are listed here for ADNI (https://ida.loni.usc.edu/collaboration/access/appLicense.jsp) and HABS (https://habs.mgh.harvard.edu/researchers/request-data/). Supplementary material is available online. Source data is included with this paper. Additional information can be provided upon reasonable request.

Field-specific reporting

Please select the one below that is the best fit for your research. If you are not sure, read the appropriate sections before making your selection.

☒ Life sciences ☐ Behavioural & social sciences ☐ Ecological, evolutionary & environmental sciences

For a reference copy of the document with all sections, see nature.com/documents/nr-reporting-summary-flat.pdf

Life sciences study design

All studies must disclose on these points even when the disclosure is negative.

Sample size	For this retrospective cross-sectional study, a priori sample size calculations for power analysis were not performed. Here, we collected our samples from established data sets (Alzheimer's Disease Neuroimaging Initiative (ADNI) and Harvard Aging Brain Study (HABS) cohorts) by selecting participants that met the following criteria: we only included participants with a 18F-flortaucipir (tau) PET and 18F-FDG PET performed within one year of each other who also had a measure of amyloid status (18F-florbetapir or 18F-florbetaben or 11C-Pittsburgh compound B or CSF amyloid-beta 42/40 ratio test) and a magnetic resonance imaging (MRI) scan (within about 1 year of PET scans). From the ADNI cohort database (http://adni.loni.usc.edu), 289 participants with a diagnosis of mild cognitive impairment (MCI) or dementia were found. In the HABS cohort (data release 2.0; access date 11/2021; https://habs.mgh.harvard.edu/), we included 115 participants with tau PET, 18F-FDG PET, 11C-Pittsburgh compound B (amyloid) PET and MRI with the same criteria as above.
Data exclusions	Data was excluded if the participant had 18F-flortaucipir (tau) PET and 18F-FDG PET scans further from 1 year from each other, and/or if no MRI or amyloid measure was available.
Replication	Internal replication of ADNI clustering results was performed 10 times by fold validation experiments. We repeated our clustering over 10 folds on 150 randomly selected participants and found similar results (see Figure S6). External replication of ADNI clustering results was performed once by evaluating with the HABS dataset, which found similar regional distributions, cognitive profiles and copathology-associated biomarker patterns as in the original ADNI dataset (see Figure S7). See the Methods and Results sections for further details.
Randomization	Randomization was not used in this observational study.
Blinding	During data acquisition and processing, the personnel performing these steps were not aware of the participants' clinical status.

Reporting for specific materials, systems and methods

We require information from authors about some types of materials, experimental systems and methods used in many studies. Here, indicate whether each material, system or method listed is relevant to your study. If you are not sure if a list item applies to your research, read the appropriate section before selecting a response.

Materials & experimental systems

n/a	Involved in the study	
☒	☐	Antibodies
☒	☐	Eukaryotic cell lines
☒	☐	Palaeontology and archaeology
☒	☐	Animals and other organisms
☐	☒	Human research participants
☒	☐	Clinical data
☒	☐	Dual use research of concern

Methods

n/a	Involved in the study	
☒	☐	ChIP-seq
☒	☐	Flow cytometry
☐	☒	MRI-based neuroimaging

Human research participants

Policy information about studies involving human research participants

Population characteristics	Population characteristics were utilized as covariates to assess modifiers of PET imaging markers. See Supplementary Tables 1 and 3 for characteristics of ADNI and HABS cohorts. These population measures and covariates included sex (female or male), age (years), education (years), amyloid status (binary A+/A–) and tau SUVR in the inferior temporal gyrus (continuous), a representative region where tau pathology correlates with disease severity. Analyses of longitudinal cognition also incorporated baseline cognition at time of 18F-FDG PET scan (such as a baseline measure of ADAS-Cog, CDR-SOB or MMSE). These characteristics were reported from ADNI and HABS data spreadsheets (see Data availability section for more information). The study samples consisted of 289 ADNI participants and 115 HABS participants. The PET data from the ADNI cohort was based on cognitively impaired participants, while the HABS cohort was for cognitively normal individuals.
Recruitment	Recruitment was performed by the ADNI and HABS programs. See the ADNI (http://adni.loni.usc.edu) and HABS (https://habs.mgh.harvard.edu/) websites for further information.

March 2021

Ethics oversight

For the ADNI data, human subjects approval was obtained by the ADNI investigators to comply with the Institutional Review Board at each participating ADNI site. All participating ADNI sites received approval from their site's Institutional Review Board; a complete listing of ADNI sites is provided at the end of the article file. All ADNI participants provided written informed consent. ADNI data was accessed according to the policies of the ADNI data sharing and publications committee. For the HABS data, HABS protocols were approved by the Partners Human Research Committee, the Institutional Review Board for the Massachusetts General Hospital and Brigham and Women's Hospital, and all participants gave informed consent. HABS data was accessed according to the policies of the HABS data committee.

Note that full information on the approval of the study protocol must also be provided in the manuscript.

Magnetic resonance imaging

Experimental design

Design type

Magnetic Resonance Imaging (MRI) data was utilized for co-registration and comparative analysis of Positron Emission Tomography (PET) imaging data.

Design specifications

Each ADNI site had different MRI/PET scanners. Detailed specifications on neuroimaging can be found on the ADNI website (http://adni.loni.usc.edu/data-samples/data-types/).

Behavioral performance measures

Not applicable; structural MRI data as obtained.

Acquisition

Imaging type(s)

structural MRI

Field strength

3T

Sequence & imaging parameters

T1-weighted (accelerated MPRAGE) and FLAIR sequence MR imaging. Imaging parameters for MRI and PET can be found on the ADNI website (http://adni.loni.usc.edu/data-samples/data-types/).

Area of acquisition

Whole brain

Diffusion MRI ☐ Used ☒ Not used

Preprocessing

Preprocessing software

ADNI MRI data was obtained with a T1-weighted structural scan (resolution 1.0×1.0×1.2mm3) and fluid attenuated inversion recovery (FLAIR) sequence MRI scan were acquired in the same session. Processed PET images with uniform isotropic resolution (8mm full-width-at-half-maximum) were obtained with the ADNI archive description “Coreg, Avg, Std Img and Vox Size, Uniform Resolution.”

Normalization

ANTs processing was performed by the Methods sections on "Imaging Data" and "Image Processing and PET Regional Analysis."

Normalization template

MRI studies were processed using the ANTs pipeline for brain extraction, template registration and cortical thickness measurement. A publicly available template constructed using cognitively normal participants from ADNI was used and can be found here (https://github.com/ntustison/CrossLong).

Noise and artifact removal

MRI studies were processed using the ANTs pipeline for inhomogeneity correction.

Volume censoring

Not applicable.

Statistical modeling & inference

Model type and settings

Not applicable.

Effect(s) tested

See the Methods section on "Statistical Analysis." Region-of-interest (ROI) measures of PET standardized uptake value ratios (SUVRs) and MRI-derived cortical thickness were compared with likelihood ratio tests across groups with covariates such as sex, age, education, amyloid status (A+/A–) and tau SUVR in the inferior temporal gyrus. Pairwise comparisons between canonical and non-canonical groups were also performed using covariates as above.

Specify type of analysis: ☐ Whole brain ☒ ROI-based ☐ Both

Anatomical location(s)

Participants' brains on MRI scans were divided into 104 cortical, subcortical, brainstem and cerebellar regions-of-interest (ROIs) with multi-atlas segmentation (http://neuromorphometrics.com/ParcellationProtocol_2010-04-05.PDF).

Statistic type for inference
(See Eklund et al. 2016)

ROI-based analysis.

Correction

See Methods section on "Statistical Analysis." Multiple tests adjustment by Benjamini-Hochberg correction was applied.

Models & analysis

n/a	Involved in the study
☒	☐ Functional and/or effective connectivity
☒	☐ Graph analysis
☒	☐ Multivariate modeling or predictive analysis
