# Supplementary material for: Dissociation of tau pathology and neuronal hypometabolism within the ATN framework of Alzheimer’s disease
Source: Nat Commun. 2022 Mar 21;13:1495. doi: 10.1038/s41467-022-28941-1 (PMC8938426; doi:10.1038/s41467-022-28941-1)
Supplement: Supplementary file 3 — Description of Additional Supplementary Files [file 41467_2022_28941_MOESM3_ESM.pdf]

Title: Supplementary Data 1
Description: Provides sample sizes and *P* values for statistical tests performed in figures and tables from the manuscript and supplement.
